# Biological potential and mechanisms of Brain-Expressed X-linked family proteins in cancers: an updated review

**DOI:** 10.1016/j.jare.2025.07.034

**Published:** 2025-07-20

**Authors:** Pingping Wang, Ziyan Chen, Hongyan Zhang, Yandan Lu, Licheng Zhou, Chenghang Gong, Dongyang An, Xianan Sang, Kuilong Wang, Min Hao, Gang Cao

**Affiliations:** a School of Pharmaceutical Sciences, The First Affiliated Hospital of Zhejiang Chinese Medical University, Hangzhou, 310053, China; b Zhejiang Cancer Hospital, Hangzhou, 310022, China

**Keywords:** BEX family proteins, Disease and cancer, Signal path, Action mechanism, Treatment strategy

## Abstract

**Background:**

Brain-Expressed X-linked (BEX) family proteins is a relative younger protein family in human being, with limited understanding of its pathophysiological functions in health and disease. So far, The human BEX family consists of five members including BEX1-5, which have highly homologous and different subcellular localization, expression patterns and proteasome degradation. Previous research found that BEX family proteins play an important role in p75NTR signal pathway which closely connected with neuron differentiation and regeneration. In recent ten years, more and more studies have shown that BEX family proteins participate in multiple molecular signaling pathways in cancers, liver diseases, heart diseases et al. These evidences hint that BEX family proteins maybe potential therapeutic targets for multiple diseases.

**Aim of review:**

In this review, we systematically summarize the research progress on the role of BEX family proteins in patients with various cancers. Our goal is to provide new treatments and insights into human cancers. In addition, the underlying mechanisms and potential clinical applications of BEX family proteins involved in various cancers were highlighted.

**Key scientific concepts of review:**

The BEX family proteins plays an important role in a variety of biological processes, including inhibiting the proliferation and metastasis of cancer cells, and participating in various biological processes such as cell differentiation, cell proliferation, and cell apoptosis.

## Highlights

• The BEX family proteins are involved in a variety of biological processes, including inhibiting the proliferation and metastasis of cancer cells.
• The BEX family proteins is involved in the occurrence of many diseases, such as breast cancer, glioblastoma, liver cancer and so on.
• The study reveals the unique and important role of the BEX family proteins in the pathogenesis of cancer.
• This review summarizes the mechanism of action and research progress of BEX family proteins in related cancers in recent years.

## Graphical abstract

**Figure:**
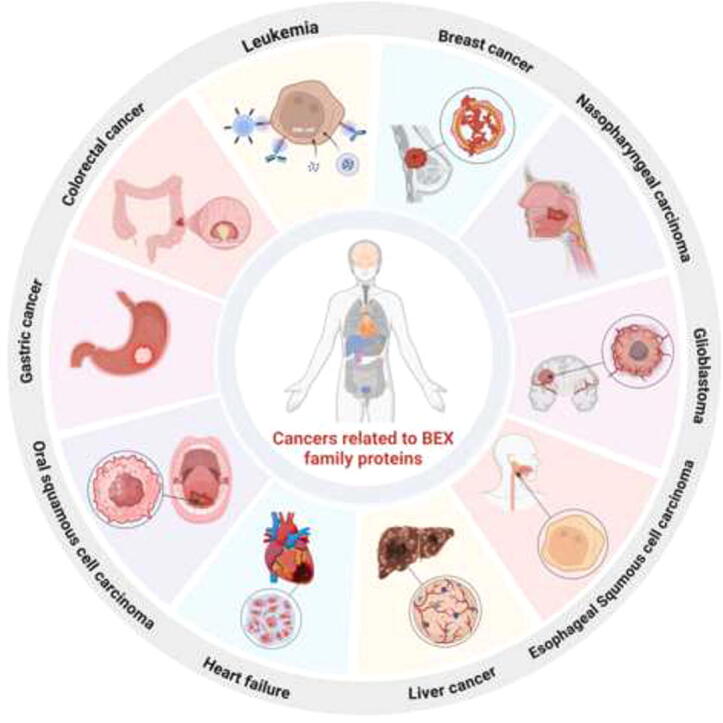

## Introduction

**Fig. 1:**
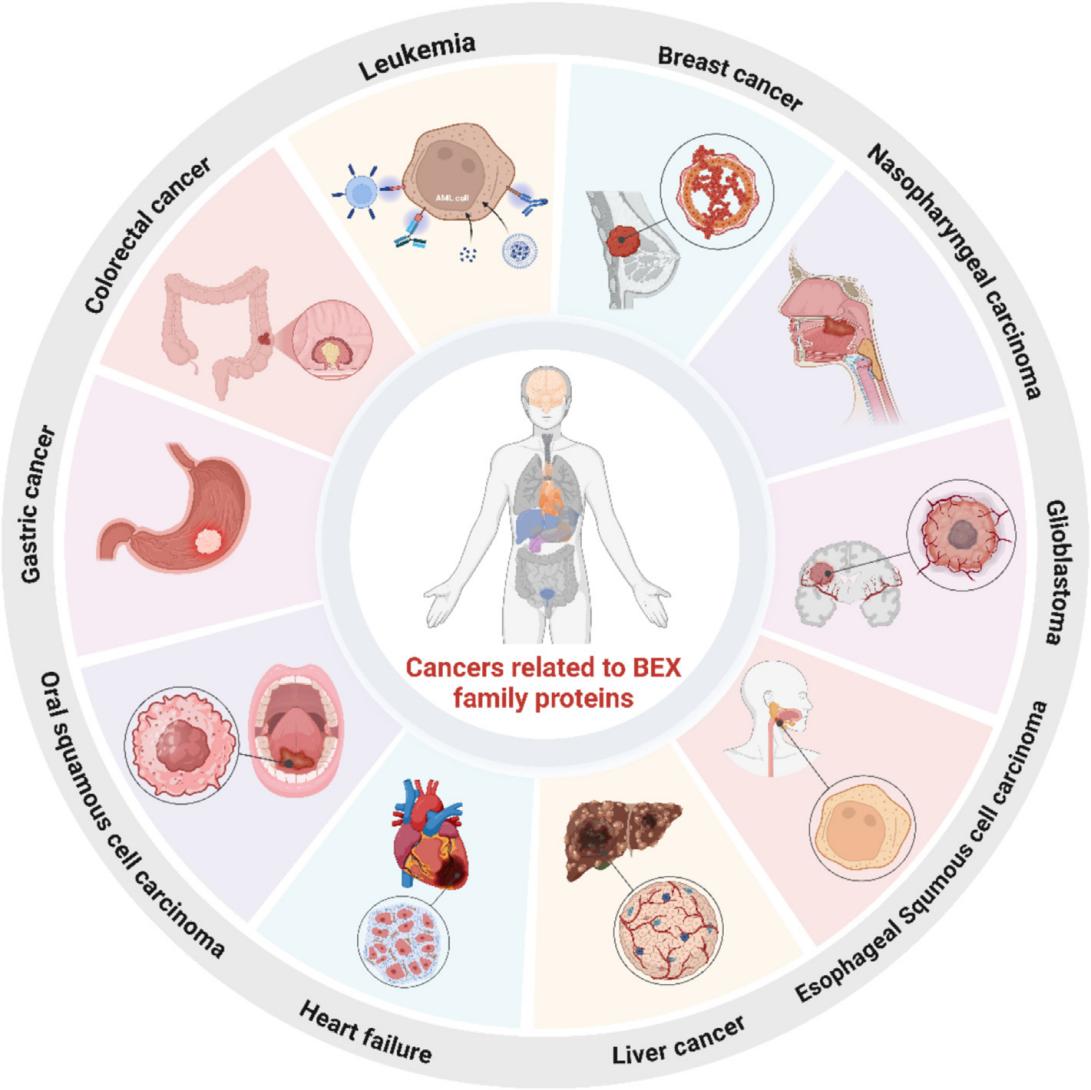
Cancers closely related to the BEX family proteins.

Molecular signaling from extracellular stimulant, such as various cytokines, give rise to kinds of cellular responses containing cell proliferation, differentiation, cell death et al. [1]. Signals transmitted by cell surface receptors were strictly regulated by intracellular proteins. In addition, various mechanisms can be employed by intracellular proteins to either enhance or impede the downstream signal transduction of receptors. The BEX family proteins, known for its ability to regulate receptors derived from various cellular surfaces, stands out as an innovative group of proteins [2]. The BEX family proteins is involved in important physiological and pathological processes such as development and differentiation, apoptosis and tumor formation. Studies have shown that Bex protein plays a role in cell cycle, cell differentiation and apoptosis. At the same time, the BEX family proteins is associated with a variety of diseases, including tumors and neurological disorders [3]. In oral squamous cell carcinoma (OSCC), the expression of BEX1 and BEX4 is correlated with the degree of tumor differentiation, lymph node metastasis and clinical stage, and may be a key factor in the clinical diagnosis and prognosis of OSCC. BEX1 has been found to play an important role in brain tumor, cerebrovascular disease, nerve injury, amyotrophic lateral sclerosis and other neurological diseases [4]. In addition, BEX proteins have been implicated in diseases such as breast cancer, glioma, acute myeloid leukemia (AML) and chronic myeloid leukemia (CML) [5] (Shown in Fig. 1). To sum up, the BEX family proteins has become a focus of research due to its multi-functionality in cell physiology and pathology, and its role in tumorigenesis and development has received particular attention. Further studies will help reveal the specific mechanisms of the BEX family proteins in the human cancers, providing new targets for clinical diagnosis and treatment.

**Fig. 2:**
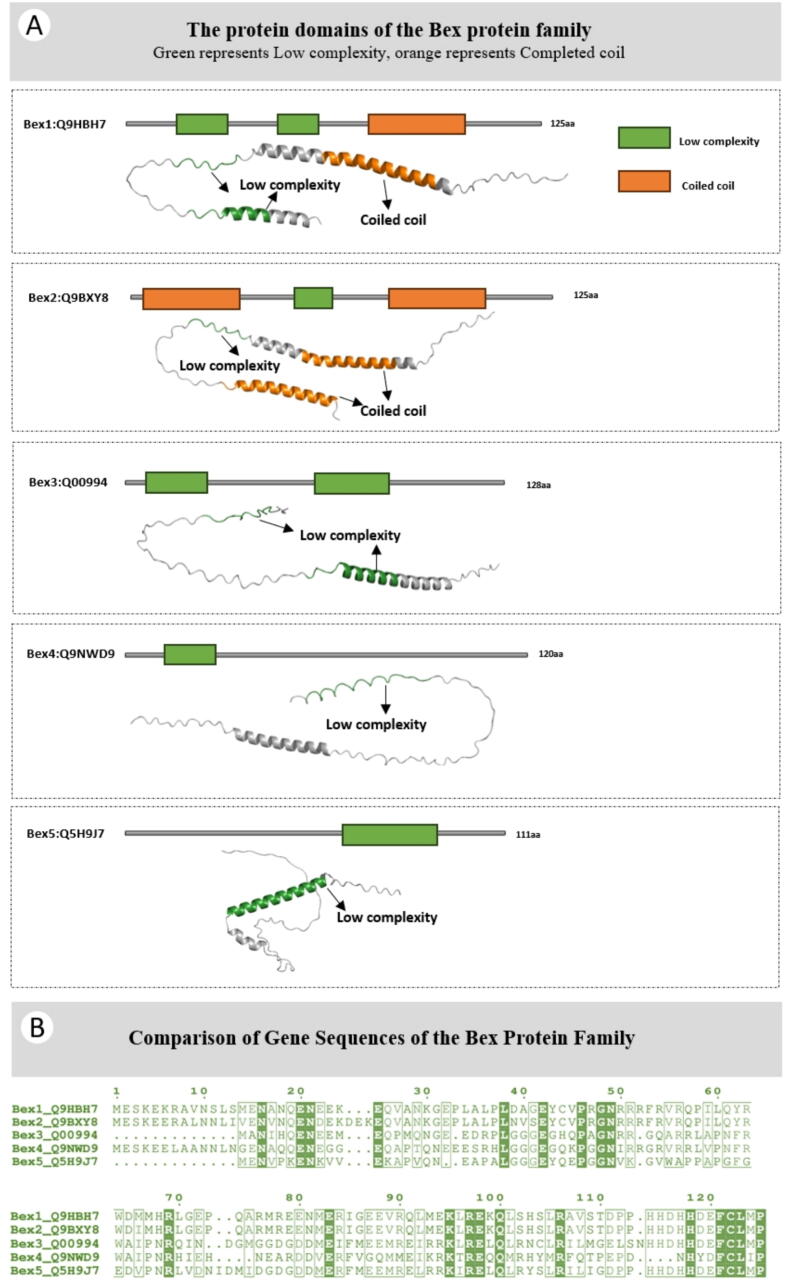
The structural domains and comparison of gene sequences of the BEX family proteins.

## BEX family proteins

Brain Express X-Linked (BEX) is an X chromosome-linked gene family, which is also a small molecule family (100-130 residues in length) . The BEX family of proteins, which are found in the central nervous system, have been shown to be crucial for a range of functions such as neuronal regeneration and differentiation [3]. With the progress of scientific research, it has been revealed that BEX family proteins exhibit extensive expression across different tissues and perform a range of functions. At the current moment, the BEX family encompasses a collective of five members known as BEX1-5. Evidence suggests that the mouse lacks a human BEX5 homolog, whereas BEX6 has been identified as a gene specific to mice and is absent in humans [6]. Members of the BEX gene family differ in subcellular localization, expression patterns, and proteasome degradation across a range of human tissues [7]. The five family members are located on the X chromosome, with BEX1, BEX2, and BEX5 positioned on the negative strand, while BEX3 and BEX4 are situated on the positive strand. In mammals, BEX family genes are very conservative [8]. In terms of structure, there is significant sequence similarity observed among various species in BEX proteins. Additionally, all BEX proteins possess a distinct BEX domain. However, due to limited research on the structural aspects of the BEX family proteins, Smart database predictions were utilized to identify protein domains. These predictions revealed that both BEX 1–5 contain a low complexity domain (LCD) (Fig. 2). Despite their lack of stable morphological distinct 3D shapes, literature reports indicate that LCDs are widely present and serve multiple biological functions. For example, they enable alterations on the terminals of all 75 intermediate filament proteins found in mammals, inhabit the central passage of nuclear pores, and embellish almost all RNA-binding proteins. In addition, LCDs have been found related with human neurological diseases [9]. In addition, the predictions suggested a strong likelihood of the presence of a coiled coil domain in BEX1-2, potentially facilitating dimerization [10].

(Fig. 2 A: The protein structures and protein 3D structures of the BEX family proteins 1–5 are respectively presented. Taking BEX1 as an example, human BEX1: Q9HBH7, with a length of 125aa, is represented by the green part representing Low complexity, and the orange part representing Coiled coil. B: Comparison diagram of 1–5 human gene sequences for the BEX family proteins) .

### BEX1

BEX1 (also known as Rex3) was first identified and cloned in the down-regulated gene of F9 teratoma cells differentiation which induced by retinoic acid [11]. BEX1 gene was located near Pyridoxal Phosphate (PLP) on mouse X chromosome. According to the corresponding relationship between human and mouse chromosomes, BEX1 gene was located at Xq22 on human X chromosome [12]. It was found that BEX1 was also expressed in the blastocyst of mouse embryos [13]. Western blot analysis revealed differential expression of BEX1 across various tissues, with elevated levels observed in the brain, testis, ovary, and pancreas. Conversely, reduced expression was detected in the heart, liver, kidney, spleen and spinal cord among others [14]. Prior studies have suggested that the involvement of BEX1 is essential in facilitating muscle differentiation [15]. Studies have shown that BEX1 is an interacting factor of p75NTR and is associated with neurotrophic factors and cell cycle. To some extent, BEX1 can act as an upstream modulator of receptor signals [16]. Peripheral axon injury can lead to overexpression of BEX1 protein, which antagonizes the inhibition of myelin-related glycoprotein on axon growth. BEX1 is involved in axon regeneration and may be a gene related to regeneration [17].

### BEX2

Prior studies have suggested that BEX2 is essential in the mechanism of muscle development [18]. In addition, the cerebellum, pituitary gland, and temporal lobe exhibit a significant expression of BEX2 [7]. Evidence suggests that BEX2 plays a crucial role in the precise control of embryonic development, potentially associated with Xq22 deletion syndrome [19]. Chunyu Han and colleagues discovered that during embryonic development, human BEX2 has the ability to specifically regulate LMO2 interaction, unlike mice BEX1 and BEX2 [20]. Furthermore, recent research has suggested that BEX2 is not solely associated with disorders of the nervous system, but also plays a significant role in the functioning of the lungs [21].

### BEX3

BEX3, initially designated as HGR74, is detected in the granulosa cells of the human prostate, testes, and ovaries [22]. Meanwhile, Human NADE is identical to HGR74/BEX3 [12]. Another literature reported that BEX3 is also highly expressed during mouse embryonic development [23]. In F9 teratocarcinoma cells, BEX3 is associated with the replication of mitochondria around the nucleus and may participate in the growth inhibition of F9[24]. Katia M. S. Cabral et al. characterized the structure of BEX3 using biophysical experimental data. Atomic force microscopy and small angle X-ray scattering show that BEX3 forms a specific high-order oligomer, consistent with the conclusion of spherical molecules. Researchers conducted solution nuclear magnetic resonance and other techniques on the recombinant protein, indicating that the structure of BEX3 is composed of approximately 31 % α-helix and 20 % β-strand. The chain consists of partially folded regions near the N and C ends, and a core with anti-proteolytic activity around residues 55–120 [25]. In the company of minute fragments of transfer RNA (tRNA), the three-dimensional structure of human BEX3 (hBEX3) becomes evident. The involvement of TRF is crucial in facilitating the transition of hBEX3 from a disordered state to an ordered state, as well as in modulating its phase [26]. BEX3′s interaction with neurotrophic factor p75NTR has been reported to be involved in P75NTR-mediated signal transduction and is known as an enforcer of p75NRT associated cell death (Nade) [27]. Initially, the association between BEX3 and p75NRT was identified using yeast two hybrid screening, which was subsequently validated through extensive in vitro and in vivo investigations [28].

### BEX4 and BEX5

BEX4 is predominantly found on the X chromosome and exhibits significant expression levels in skeletal muscle, heart, and liver tissues [7]. Up-regulation of BEX4 will affect the cell growth characteristics and protein expression profile, and may be involved in the testicular injury caused by cadmium chloride [29]. BEX4 gene is located in the spindle pole and microtubule, and its expression can resist cell apoptosis and increase tumor growth and proliferation. Research has shown that BEX4 is associated with oral cancer, acting as a tumor inhibitor by inhibiting the proliferation and growth of oral cancer [4]. The sequence of BEX5 is similar with BEX4. At present, there is relatively little research on BEX5 both domestically and internationally.

## BEX family proteins interacting proteins

**Fig. 3:**
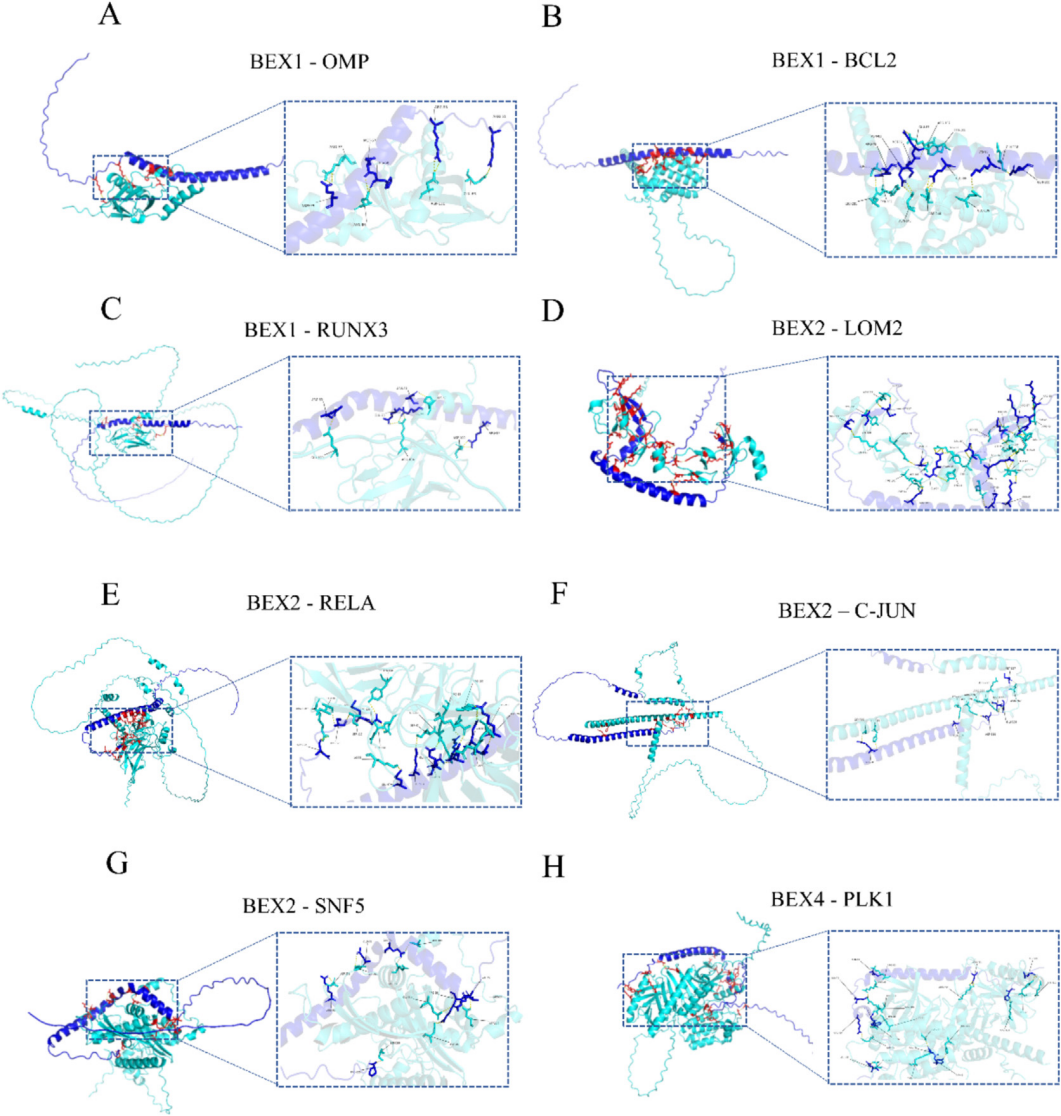
Molecular docking diagram of BEX family proteins and interacting proteins.

Currently, many studies have shown that the BEX family proteins has interacting proteins. Intrinsically disordered proteins (IDPs) constitute the key points of network regulation, which are abundant in complex organisms [30]. It is reported that BEX protein constitutes new IDPs [2]. Emi Hibino et al. demonstrated that BEX1 exhibits the characteristic physical and chemical properties of an intrinsically disordered protein (IDP), thereby playing a crucial role in facilitating tubulin polymerization as a reaction site for the formation of primary cilia [31]. The presence of olfactory marker protein (OMP) serves as a clear indication that olfactory receptor neurons (ORN) have reached a mature state in all vertebrate species [32]. In the study by JAE HYUNG KOO et al., it was found that BEX1 and 2 were identified as interaction partners of OMP. The article delves into the distribution of BEX1 and BEX2 mRNA in different regions of the brain, as well as the development and characterization of mouse antibodies that can recognize both BEX1 and BEX2 proteins. These antibodies were utilized to determine the cellular localization pattern of BEX proteins [33]. Furthermore, the dimeric form of OMP is indispensable for facilitating this interaction with BEX1/2 in vivo. Given its short half-life and specific subcellular localization, it strongly implies that the BEX/OMP dimer complex plays a pivotal role in the entire olfactory transduction cascade mediated by OMP [34]. OMP mRNA co-localizes with BEX1, BEX2, and BEX3 mRNA in the ORN of mice. The interaction between OMP and BEX is confirmed through chemical crosslinking experiments [35]. In addition, there are also some proteins that interact with the BEX family proteins, among which the RUNX protein, as the main participant in the pathogenesis of cancer, has quickly gained the attention of researchers. Three members of the RUNX family have been identified in mammals [36]. In the research results of Qian Wang et al., it was found that blocking the interaction between BEX1 and RUNX3 has a significant impact on β-catenin inhibition of transcription, thereby activating Wnt/ β- Catenin signaling and maintaining the dryness of hepatocellular carcinoma (HCC) and Hepatoblastoma (HB) with high cancer stem cell (CSC) features [37].Compared with normal cells, anti-apoptotic protein BCL-2 is a protein that is highly up-regulated in many cancers, which makes it an ideal target for cancer treatment [38]. Qian Xiao and colleagues discovered the association between B-cell lymphoma 2 (BCL-2) and BEX1. The prevention of the formation of anti-apoptotic bcl2/bcl2-related X protein was observed when inhibiting the interaction between BCL-2 and BEX1, thereby enhancing Imatinib-induced apoptosis [39]. Koo et al. compared the BEX1 gene knockout (BEX1 ko) mice with wild type (WT) mice, and found that the gene knockout mice had functional defects in exercise capacity. Through research, they found that BEX1 combined with CaM in a calcium dependent manner, providing evidence of changes in muscle regeneration of BEX1 gene knockout mice. The interaction between BEX1 and calcium/calmodulin may participate in skeletal muscle regeneration [40]. Han Qiuyue et al. discovered that INI1/hSNF5 functions as a binding protein of BEX2, as determined by screening with a yeast two-hybrid system in which BEX2 serves as the “bait” protein. The article further confirms the direct and specific interaction between BEX2 and INI1/SNF5 through GST Pull down experiments. Subcellular localization analysis reveals predominant nuclear distribution for both BEX2 and INI1/SNF5. Further exploration of the interaction between the two revealed that BEX2 affects the cell cycle through its interaction with INI1/SNF5 [41]. Chunyu Han and colleagues have established the specific interaction between LMO2, a transcription factor containing the LIM domain, and BEX2 using GST BEX2 pull-down and in vivo co-immunoprecipitation assays. They have also shown that in vivo, BEX2 has the ability to increase the transcriptional activity of LMO2 [20]. Ali Naderi et al BEX2 has functional interactions with p65/RelA and c-Jun in breast cancer. In this process, BEX2 is a target gene for p65/RelA and c-Jun, which can regulate the phosphorylation/activity of these proteins [42]. PLK1, a kinase that acts on serine and threonine residues, plays essential roles in diverse cellular processes such as cell division, apoptosis, and response to DNA damage. Literature reports indicate that the interaction between PLK1 and BEX4 can induce aberrant mitotic cells to adapt to aneuploidy instead of undergoing programmed cell death [43]. Consequently, BEX4 has been recognized as a newly discovered oncogenic protein in human malignancies [44]. These interacting proteins reveal the role of BEX1 in mRNA processing, translation, and basic microtubule transport. These findings provide new insights into the function of BEX1 within cells and may have important implications for the study of diseases such as cardiovascular disease and cancer. The molecular docking of BEX family proteins with their interacting proteins is shown in Fig. 3.

## BEX family in cells

Cells are the fundamental units of human physiological activities, and the complex signaling mechanisms in various cells significantly affect human health and disease status. More and more literature reports have found that the BEX family proteins plays different regulatory roles in different types of cells. In depth research on the signaling regulation mechanism of this protein family in cells may bring new ideas for solving disease treatment strategies.

### BEX in cell death

Cell death is the most basic feature of the development of all multicellular organisms. The investigation into cellular apoptosis will have a profound impact on the management of various medical conditions [45]. Himakshi Sidhar and his colleagues found that curcumin can upregulate BEX1,2,3,4 in a time-dependent and dose-dependent manner, inducing N2a cell apoptosis. PI3K, JNK, and P53 inhibitors can block this process. Further research has found that curcumin can activate the P53 ser-15 phosphorylation site and upregulate the BEX gene family to promote apoptosis [46]. Justin Judd et al. found that BEX1 has been identified as a factor that not only inhibits cell apoptosis but also enhances DNA replication. It participates in the regulation of cell growth and programmed cell death throughout the development and maturation of myocardial cells [47]. Ferroptosis belongs to non-apoptotic regulation of cell death and is related to many diseases [48]. The researchers discovered that BEX1 has the ability to inhibit ferroptosis in vitro, specifically in cultured human adrenal cortical cells. This suppression ultimately enhances the viability of adrenal cells, suggesting a potential involvement of BEX1 in the development of aldosterone adenoma [49]. Research conducted by Jeong Ae Park et al. revealed the involvement of BEX3 in neurodegeneration that is dependent on Zn2 + and its contribution to cellular apoptosis [50]. Jin-Kwan Lee et al conducted a series of experiments in vivo/vitro which manifested that the high expressions of BEX4 can enhance the multiplication potential and growth of tumors by transfer apoptotic cell fate from death to aneuploidy. The extensive examination of the molecular mechanism uncovered a direct association between cancer-causing potential and increased levels of BEX4 expression, leading to enhanced acetylation of α-TUB by impeding SIRT2-mediated deacetylation [51].

### BEX family in cancer cells

TAKEFUMI DOI team was able to identify the invasion and proliferation of A110L and MSTO-211H cell lines by using the bilayer collagen gel hemispherical (DL-CGH) method, resulting in a significant increase in BEX1 expression in the invasive cells. Furthermore, suppressing the expression of BEX1 effectively hindered the invasion and proliferation of invasive tumor cells. This suggests that BEX1 could potentially enhance the spread of cancer cells in living organisms and may serve as a promising molecular target for addressing malignant pleural mesothelioma and LAC [52]. Promoter hypermethylation and transcriptional silencing are common epigenetic mechanisms of gene inactivation in cancer. Katherine Karakoula et al. used global expression microarray analysis to compare short-term ependymoma cells that were not treated with demethylation agents and those that were treated with demethylation agents, and found 55 potentially silencing genes, including BEX1. Further COBRA combined genome sequencing of 40 ependymoma clinical samples showed that BEX1 had the highest degree of methylation. In addition, in pediatric ependymoma short-term cell culture, ectopic expression of BEX1 significantly inhibited cell proliferation and colony formation, suggesting that BEX1 may be a candidate tumor suppressor gene (TGS) for intracranial ependymoma due to epigenetic inactivation [53]. About 40 years ago, the concept of Cancer stem cells (CSCs) was put forward, and it was pointed out that the growth of tumor is similar to that of healthy tissue, is fueled by small numbers of dedicated stem cells [54]. CSCs are subsets of cancer cells with different mechanisms leading to heterogeneity in tumors [55]. The gene BEX2 has been demonstrated to be associated with CSCs. In their study, Satoshi Saijoh et al. conducted a fusion experiment by introducing the BEX2 protein into the bile duct cancer cell line NanoLuc expressing HUCCT1. Through high-throughput screening, they identified BMPP (1,3-Benzenediol, [4-(4-methoxyphenyl)-1H-pyrazol-3-yl]) as an inhibitory factor of BEX2. The discovery of this small molecule compound and its role in CSCs overexpressing BEX2 holds significant implications [56]. The expression of BEX4 is found to be markedly elevated in LAC tissues when compared to the surrounding tissues. Experimental findings indicate that BEX4 acts as an oncogene in LAC, and its increased expression may potentially contribute to the development of LAC triggered by heightened mTOR activity [57].

### BEX family in nerve cells

Steven L. Bernstein et al. proved that the signal level of BEX was lower in the smaller retinal ganglion cells (RGC) neurons, and there was a transcription factor Brn3b that was first expressed at a higher level. In general, the evaluation of RGC cell bodies and axons distribution in the retina in vitro can be accomplished by BEX immune response, but also evaluate the influence of rodent anterior is chemic optic neuropathy (Raion) on RGC axon loss. Hence, BEX could potentially contribute significantly to assessing the impact of nerve stroke on both immediate and lasting outcomes. This is due to its potential involvement in regulating the function of cell body components within RGCs as well as influencing the axons of RGC neuron [58]. Mingqing He et al. studied the relationship between BEX1 and the pathophysiology of intracerebral hemorrhage (ICH). The findings indicated a notable increase in the expression of BEX1 within the neuronal population surrounding the hematoma following ICH. The occurrence of nerve cell apoptosis following ICH can be mitigated through the utilization of BEX1, representing a novel molecular target for pharmacological intervention in ICH treatment [59]. Peng Li's team found that BEX2-dependent autophagy can be activated by isoflavones, and isoflavones have protective effects on Atrazine (ATR)-induced neuronal apoptosis [60]. In subsequent studies, the team found that the activation and neuroprotection of BNIP3/NIX pathway induced by Soybean isoflavones (SI) could be inhibited by the deletion of BEX2 gene. These findings suggest that the BEX2/BNIP3/Nix pathway has important significance in ATR-induced neurotoxicity and mitochondrial dysfunction [61]. The team led by Laura Calvo demonstrated that the rat BEX3 protein specifically regulates the expression of TrkA at the level of its gene promoter, thereby playing a crucial role in modulating TrkA receptor expression. The down regulation of BEX3 using shRNA increases neuronal apoptosis in NGF-dependent sensory neurons deprived of NGF and compromises PC12 cell differentiation in response to NGF. In addition, BEX3 plays a crucial role in the control of nerve NGF induced activities by managing the TrkA promoter [62]. The interaction between NADE and hamartin, a protein produced by the gene tuberous sclerosis complex 1, has the potential to impact the functionality of neuronal cells [63]. Enrique Navas-P é rez et al. first established mice with BEX3 gene knockout to investigate the association between BEX3 and advanced brain function in mammalian and human neurological diseases. They found that knocking out BEX3 resulted in structural changes in the brain, cerebellum, frontal bone, and other structures. Subsequently, it was found that the mice exhibited a series of behavioral and cognitive changes, as well as electrophysiological imbalances in the hippocampus. Meanwhile, data shows that BEX3 deficiency leads to excessive phosphorylation of Akt on Ser-473 in brain extracts, indicating excessive activation of mTORC2 [64].

### BEX family in other cells

The differentiation direction and activation degree of liver progenitor cells are related to the type of damaged mature epithelial cells (hepatocytes or bile duct epithelial cells) and the severity of the disease [65]. Keiichi Ito and colleagues demonstrated that stem/progenitor cells exhibit significant expression of the BEX gene family based on their comprehensive analysis of hepatocyte genomes. The experimental findings revealed a robust association between the manifestation of BEX2 and the advancement of stem/progenitor cells, underscoring its noteworthy implications for forthcoming investigations in endocrine and stem/progenitor cell research [66]. Chunhui Jiang et al. identified that BEX1 is related to myoblast fusion. Its overexpression promotes the fusion of primary myoblasts, but does not affect the expression of myogenin expression and myoblast differentiation. BEX1 plays a new role in myogenesis by regulating myoblast fusion [67].

## BEX family proteins in cancer

### Gastric cancer

Gastric cancer (GC) is one of the most common malignancies in the world, and the development of GC is related to Helicobacter pylori infection, genetic factors, and lifestyle factors, such as alcohol consumption and smoking [68]. Due to the diagnosis of GC is generally in the late stage, its mortality is very high, with more than 1 million new cases every year [69]. BEX2 has the ability to enhance tumor stemness and cisplatin resistance and is considered a poor prognostic indicator of GC. Its expression was increased in globular cells, and BEX2 up-regulated the expression of CHRNB2, a cancer stem-related gene, and its deletion also decreased aldefluor activity. Therefore, targeting BEX2 may be a promising therapeutic strategy for controlling malignant progression of GC [70]. Zhu Chenhong et al. confirmed that the expression level of BEX4 in clinical GC samples was low. Through gene set enrichment analysis (GSEA) and human Protein mapping (HPA) database, it was found that the expression of BEX4 was related to RNA polymerase, Cell cycle, DNA replication, P53 signaling pathway, etc. BEX4 is a promising prognostic indicator for GC [71].

### Oral squamous cell carcinoma

Oral squamous cell carcinoma (OSCC) occurs on the mucous epithelium of the mouth and is a common type of cancer affecting the head and neck. It accounts for about 90 % of oral malignancies and impairs appearance, pronunciation, swallowing and taste. OSCC shows a significant incidence, but survival outcomes are inadequate [72]. Lee et al. identified BEX1 and LDOC1 as two epigenetic silenced X-linked tumor suppressor factors through high-throughput screening methods. Methylation of BEX1 and LDOC1 promoters was significantly associated with OSCC, and OSCC cells overexpressing BEX1 and/or LDOC1 exhibited strong in vitro and in vivo growth inhibition effects. This is because the expression of p50 and p65 is reduced, and the restored expression of BEX1 and LDOC1 inhibits the NF − κB signaling pathway [73]. In addition to BEX1, BEX4 has also been reported to be involved in the development of OSCC. After the administration of trichostatin A and Zebularine, BEX4 expression was reactivated in cell lines of OSCC. The presence of BEX4 significantly contributes to the inhibition of growth and proliferation in OSCC [4].

### Nasopharyngeal carcinoma

Nasopharyngeal carcinoma (NPC) is a relatively uncommon form of cancer that arises from the epithelial cells lining the mucosa of the nasopharynx [74]. The main contributing factors of NPC are environmental changes and lifestyle [75]. Wei Gao and colleagues performed a meta-analysis using microarray technology to investigate the expression profiles of the BEX gene family in various types of cancer, aiming to gain insights into their roles in human malignant tumors. The findings indicated that BEX3 exhibited no upregulation in other forms of cancer, with its upregulation being restricted solely to head and neck malignancies. It assumes a distinctive function in the development of nasopharyngeal carcinoma [76]. CD271 is a candidate stem cell manufacture for head and neck cancer. In Wei Gao et al. 's study, BEX3 is a CD271 receptor-related protein that is overexpressed in NPC. In cisplatin-resistant NPCS, high expression of BEX3 is associated with high expression of octamer-binding transcription factor 4 (OCT4), and inhibition of BEX3 expression can significantly reduce OCT4. Targeting BEX3 with shRNA can improve the sensitivity of nasopharyngeal carcinoma cells to cisplatin [76].

### Colorectal cancer

**Fig. 4:**
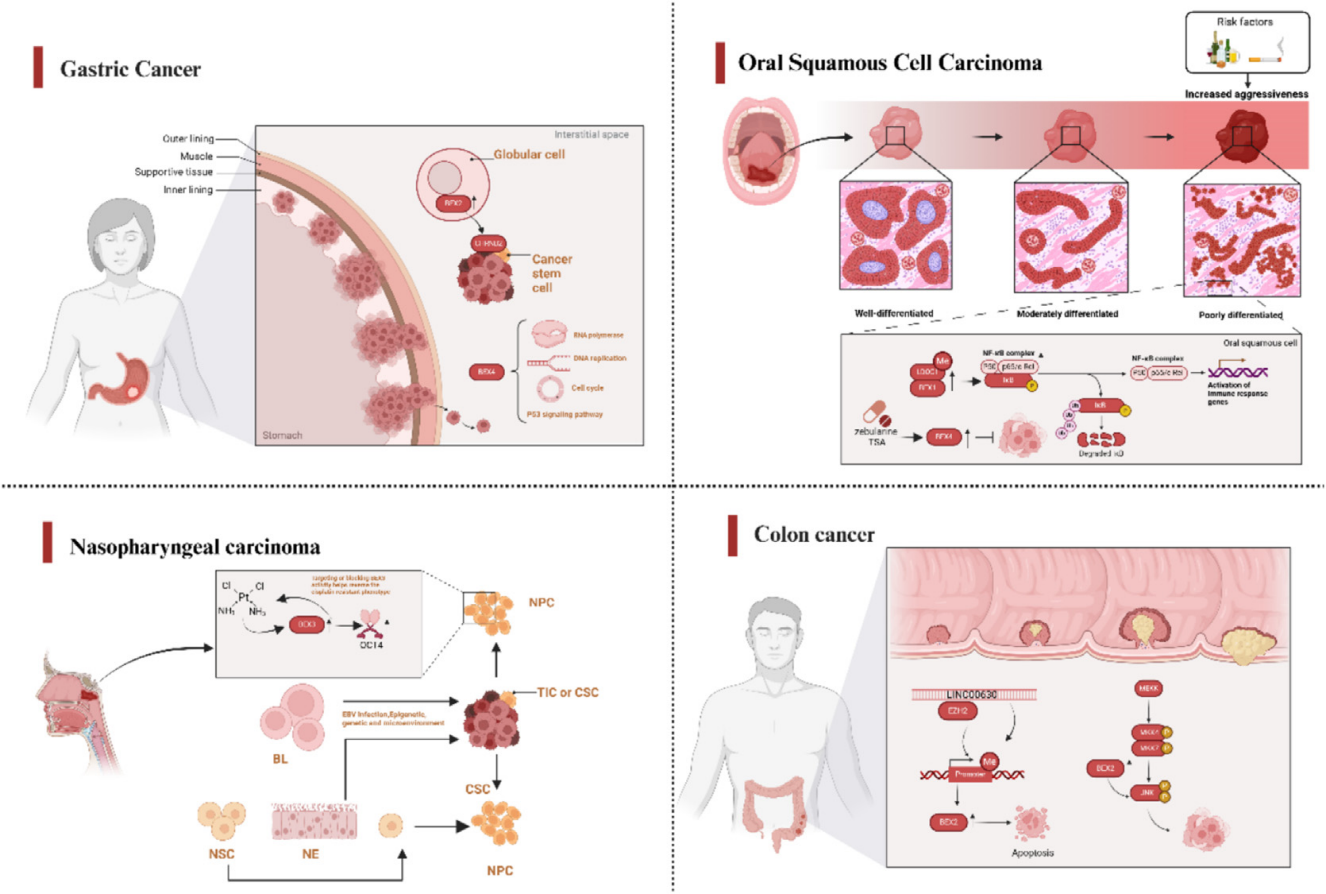
Mechanism diagram of BEX family proteins related to GC, OSCC, NPC and CRC. (Fig. 4: BL, Blymphocvtes; CSC, cancer stem-like cell; EBV, Epstein-Barr virus; NE, nasopharyngeal epithelial cells; NPC, nasopharyngeal carcinoma; NSC, normal stem cell; TIC, tumor-initiating cell.) .

Colorectal cancer (CRC) is the third most common cause of cancer worldwide, with an average annual mortality rate of approximately 900,000 people. Despite improved survival rates, metastatic CRC (mCRC) remains a fatal disease with a 5-year survival rate of approximately 14 % [77]. Eating habits, smoking, lack of exercise and obesity all increase the risk of CRC [78]. Over the past decade, a plethora of evidence has emerged highlighting the pivotal role of BEX2 in tumor carcinogenesis and its oncogenic potential across various malignant neoplasms [79]. Feng Liu et al. demonstrated that the elevation of long non coding RNA (lncRNA) LINC00630 is associated with radiation resistance and poor prognosis in CRC, with knockdown of LINC00630 significantly increasing the sensitivity of CRC cells to radiation. Simply put, the LINC00630 complex with EZH2 negatively regulates BEX1 through promoter DNA methylation, and BEX1 silencing greatly restores cell viability and inhibits apoptosis [80]. Research reports that BEX2 regulates the cell cycle through the JNK/c-Jun signaling pathway, thereby promoting CRC cell proliferation. This study demonstrates a direct association between BEX2 and rectal cancer, indicating its broad prospects as a new therapeutic target for rectal cancer [81]. In addition, Yinuo Tan et al. demonstrated that BEX2 exerts inhibitory effects on the Hedgehog signaling pathway in CRC cells by impeding the nuclear translocation of Zic2, thereby establishing BEX2 as a novel regulator of metastasis in colon cancer cells[82]. Fig. 4 shows the mechanism of the BEX family proteins associated with the above diseases.

### Glioblastoma multiforme

**Fig. 5:**
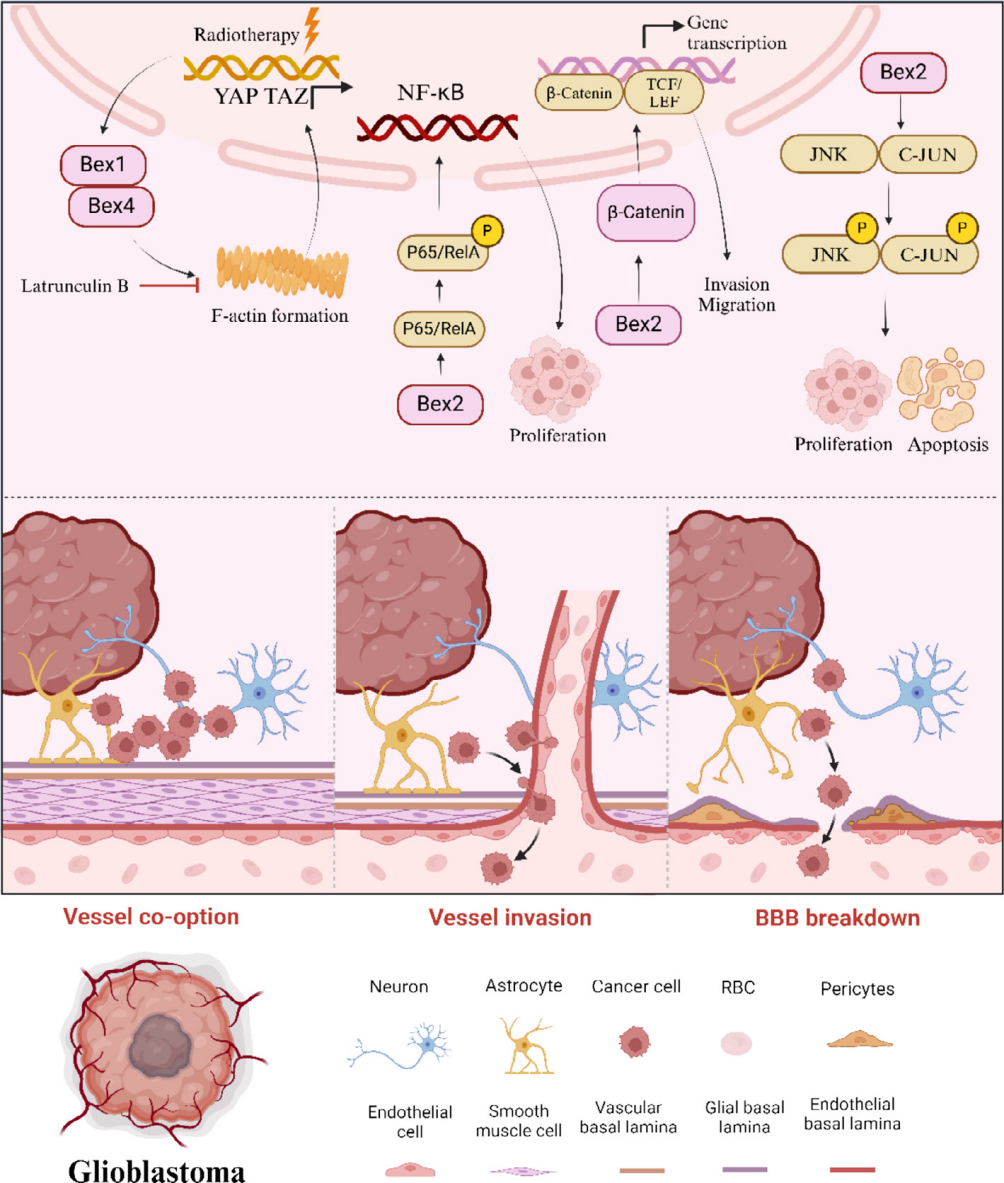
The BEX family proteins and signaling pathways associated with glioblastoma.

Glioblastoma multiforme (GBM) is recognized as the most aggressive and prevalent malignant primary brain tumor. Patients diagnosed with GBM frequently face poor prognoses, with a median survival rate of approximately 15 months [83,84]. In general, the symptoms and signs of GBM are relatively mild and tend to worsen gradually [85]. Existing research shows that LINC00526, as a sponge of microRNA-5581-3p, will eventually affect the progression of glioma through the expression of BEX1 [86]. Marie Le Mercier et al used the Hs683 cell line as an oligodendroglioma model, and galactin-1 (Gal-1) has been shown to be involved in chemotherapy resistance, new angiogenesis, and migration in Hs683 oligodendroglioma. This study demonstrates the potential role of BEX2 in the cell biology of Hs683 oligodendroglioma. The expression of Gal-1 in Hs683 cells was down-regulated by targeting small interfering RNA, resulting in a significant decrease in the expression of BEX2 [87]. The scientists created a mouse model of GBM by xenotransplantation and explored the molecular mechanism responsible for the progression of GBM following radiotherapy. Examination of gene transcription demonstrated an elevation in the levels of BEX1 and BEX4 expression within GBM cells that exhibited resilience following radiotherapy. BEX1 and BEX4 play a role in activating the YAP/TAZ signaling pathway through filamentous cytoskeleton formation and mechanical transduction, while latrunculin B inhibits GBM progression after radiotherapy by suppressing actin polymerization [88]. Overexpression of BEX2 enhances cell migration and invasion, while downregulation of BEX2 inhibits these processes. In addition, downregulation of BEX2 was accompanied by an increase in N-cadherin and a decrease in MMP-2 secretion. These results suggest that BEX2 may affect the migration and invasion of glioma through both N-cadherin and MMP-2 pathways [89]. The researchers also discovered that BEX2 has the ability to control the growth and programmed cell death of cancerous glioma cells via the c-Jun NH2 terminal kinase (JNK) pathway, These effects were eliminated by the administration of the JNK-specific inhibitor SP600125 [90]. Meng et al. investigated the main mechanism by which BEX2 promotes glioblastoma cell proliferation. Downregulation of BEX2 inhibits glioma cell proliferation and expression of NF-κB p65, but overexpression of BEX2 promotes them. Similarly, downregulation of p65 inhibited the proliferation of glioma cells, but overexpression of p65 increased the proliferation of glioma cells [91]. ErNie et al. found that downregulation of BEX2 inhibited the migration and invasion of glioma cells by reducing the expression of β-catenin in the nucleus and cytoplasm [92]. Greg Foltz et al. successfully established a new platform for screening epigenetic silencing genes of malignant gliomas in the whole genome through a comprehensive method. The tumor inhibition function of BEX1 and BEX2 genes in malignant gliomas has been confirmed [93]. Adilai Aisa et al explored the biological function and prognostic value of the BEX family in GBM by using the Cancer Genome Atlas (TCGA) database to screen for differentially expressed BEX genes between normal tissues and GBM. Univariate and multivariate Cox regression analysis identified prognostic genes BEX1, BEX2 and BEX4 involved in the regulation of immune response. Correlation analysis and protein–protein interaction network (PPI network) results showed a significant correlation between the BEX family and the TCEAL family in GBM [94]. The mechanism by which GBM are associated with the BEX family proteins is shown in Fig. 5.

### Breast cancer

**Fig. 6:**
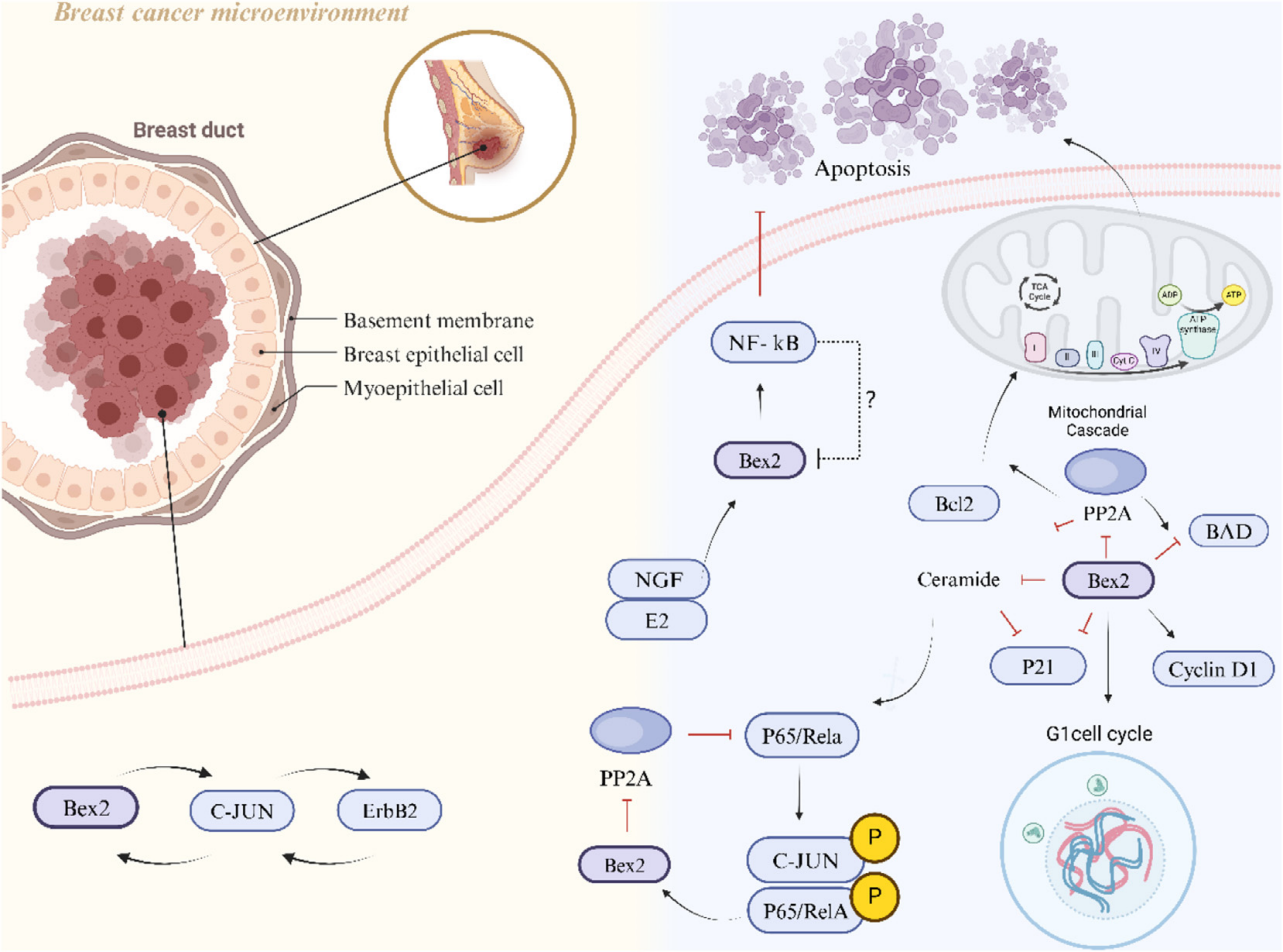
The BEX family proteins and signaling pathways associated with breast cancer.

Breast cancer is still the main cause of cancer-related deaths in women. In the past two decades, although the survival rate of breast cancer has significantly improved, the incidence rate of this disease continues to rise worldwide [95]. Breast cancer, along with lung cancer and colon cancer, is widely recognized as one of the most prevalent types of cancers globally. It predominantly affects women and is considered the most frequently occurring malignant tumor among them [96]. Evidence shows that Wnt signal activation driven by gene mutation is the key factor for breast cancer metastasis [97]. Ali Naderi et al. found that BEX2 is essential for NGF to inhibit C2-induced apoptosis, which NGF inhibits by binding activated NF-κB and p75NTR. In addition, BEX2 was found to regulate apoptosis of breast cancer cells under the action of estradiol (50 nmol/L) and tamoxifen (5 and 10 mmol/L) [98]. Ali Naderi's team observed that BEX2 exhibits varying levels of expression in breast cancer cells and has a protective effect against mitochondrial apoptosis by regulating the Bcl-2 protein family, which is involved in the positive regulation of anti-apoptotic members Bcl-2 and the negative regulation of pro-apoptotic members BAD, BAK1 and PUMA. In addition, BEX2 affects cyclin D1 and p21 to restore normal progression of the G1 phase cell cycle in breast cancer cells [99]. In later studies, the team also discovered the transcriptional regulation of BEX2 and the feedback mechanism that mediates the gene's cellular function in breast cancer. BEX2 has functional interactions with p65/RelA and c-Jun in breast cancer. In this process, BEX2 is a target gene for p65/RelA and c-Jun, which can regulate the phosphorylation/activity of these proteins. More importantly, downregulation of BEX2 can lead to a significant increase in PP2A activity in c-Jun stable lines, and these findings suggest that BEX2 is involved in a novel feedback mechanism with important implications for the biology of breast cancer [42]. The team then found that the BEX2 protein is overexpressed in about half of all breast cancers, and combined with previous studies, they identified the functional feedback loops of ErbB2, c-Jun and BEX2 in breast cancer cells by creating in vitro models. In this loop, overexpression of ErbB2 leads to expression of c-Jun and BEX2, in turn, the overexpression of BEX2 led to an increase in both c-Jun-mediated induction of ErbB2 and c-Jun binding to the ErbB2 promoter in MCF-7 cells [5]. The mechanism by which breast cancer is associated with the BEX family proteins is shown in Fig. 6.

### BEX family proteins in liver diseases

**Fig. 7:**
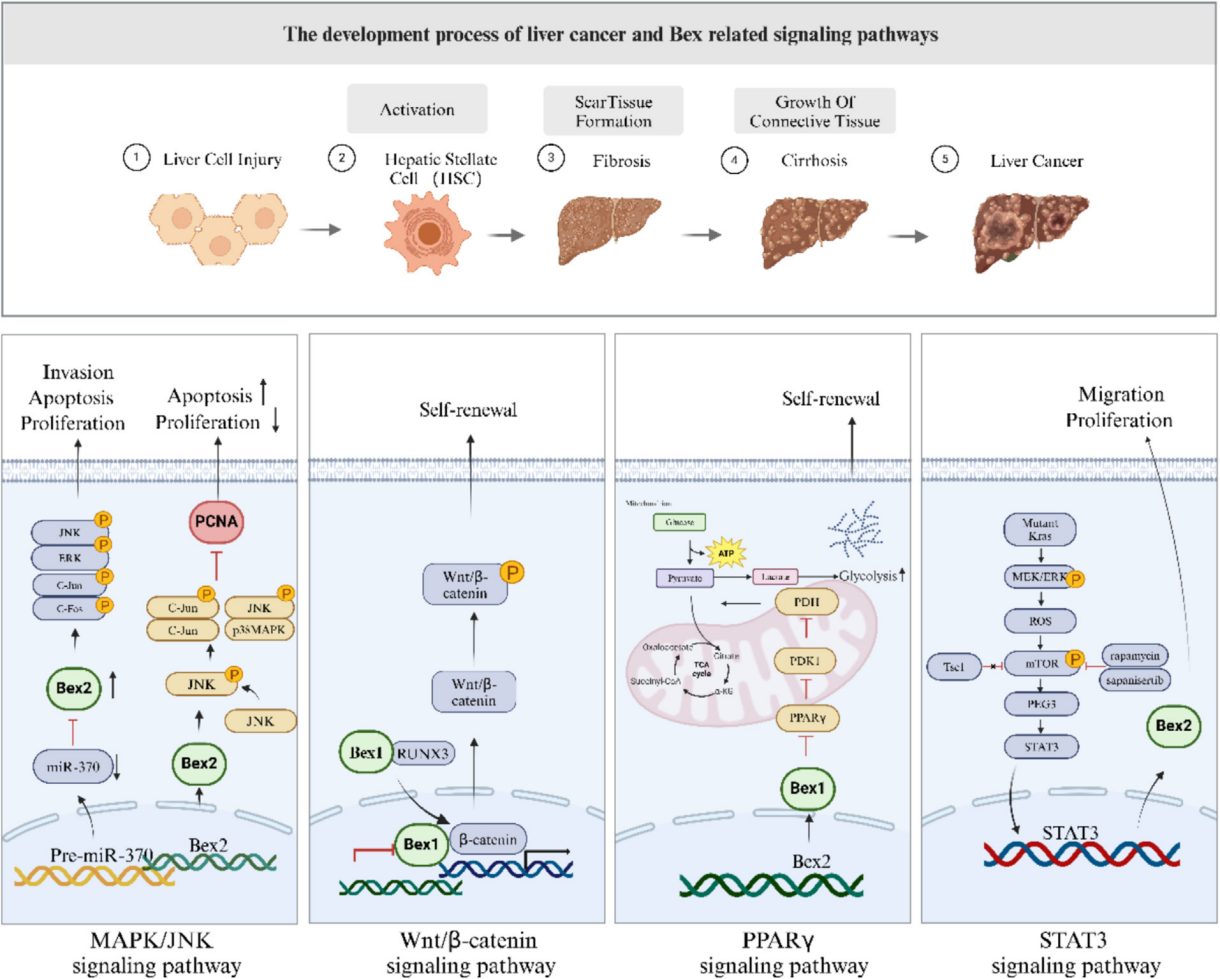
The BEX family proteins and signaling pathways associated with liver disease.

In mammals, the liver mainly plays the metabolic, secretion and transport of bile, detoxification and hematopoietic functions, and the liver has a unique and powerful repair ability to cope with a variety of injuries [100]. In recent years, there has been a rise in the occurrence of liver cancer, which mainly includes intrahepatic cholangiocarcinoma (ICCA) and HCC. Liver cirrhosis caused by hepatitis B and C and alcohol are the main causes of liver cancer [101]. In the study by Takeki Uehara et al., the significant effect of liver fibrosis induced by genotoxic factors was associated with replicative aging in non-cancerous liver tissue (such as p16 overexpression), carcinoembryonic transformation (increased expression of H19 and BEX1), and “stemness” (such as increased expression of Prom1 and Epcam) [102]. Previous research has indicated that BEX1 functions as an embryonic protein in cancer, specifically serving as a novel marker for HCC and HB. Mechanistically, DNMT1 mediates BEX1 promoter methylation and BEX1. The inhibition of β-catenin transcription is facilitated by the interaction between BEX1 and Runx3, leading to the activation of the Wnt/β-catenin signaling pathway. DNMT1 plays a role in regulating BEX1, promoting self-renewal and maintenance of hepatic CSCs through the activation of Wnt/β-catenin signaling. Consequently, targeting BEX1 could hold significant therapeutic potential for HB and CSC-HCC [37]. Qian Wang e et al have demonstrated that the overexpression of BEX1 amplifies the Warburg effect in HB cells. The mechanism behind this phenomenon involves BEX1′s ability to decrease the levels of peroxisome proliferator-activated receptor-γ (PPARγ). In HB cells, Pyruvate dehydrogenase kinase isoenzyme 1 (PDK1) plays a crucial role in inhibiting the Warburg effect induced by PPARγ. Furthermore, BEX1 contributes to maintaining stemness in HB by regulating the Warburg effect through the PPARγ/PDK1 pathway. Additionally, pioglitazone can target BEX1-mediated stemness properties in HB by increasing the expression of PPARγ. In conclusion, targeting BEX1 may hold promise as a novel therapeutic strategy for HB [103]. Na Zhuang et al. have shown that BEX1 is a key mediator of sorafenib resistance in HCC, and the expression of BEX1 is significantly reduced in sorafenib resistant HCC cells and xenotransplantation models. It was also found that the expression of BEX1 in HCC tissues was down-regulated compared with normal liver tissues in the TCGA database. Further studies showed that BEX1 sensitised HCC cells to sorafenib by inducing apoptosis and negatively regulated Akt phosphorylation [104]. Another study found that hepatocyte liver cells of mice with alcoholic hepatitis exhibit a significant increase in the expression of BEX2. BEX2 facilitates apoptosis in hepatocyte liver cells and hinders their proliferation through the activation of the JNK/MAPK pathway. Thus exacerbating the progression of acute alcoholic hepatitis [105]. Evidence reported that microRNAs play a key role in the occurrence and development of HCC, and BEX2 is highly expressed in HCC cell lines and tissues, while miR-370 is low expressed. In vivo experiments demonstrated that overexpression of miR-370 inhibited tumor growth, and BEX2 was the target of miR-370. miR-370 can down-regulate the BEX2 gene and inhibit the activation of MAPK/JNK signaling pathway, thereby promoting apoptosis of HCC cells and inhibiting their proliferation, migration and invasion [106]. Yuan Deng Luo et al. demonstrated experimentally that Kras mutants facilitate mTOR activation through ROS accumulation, thereby promoting HCC metastasis and development. Mek/Erk augments the anticancer efficacy of mTOR inhibitors by attenuating mTOR activity, while PEG3 interacts with STAT3 and enhances its transcriptional activity. In previous reports, mTOR upregulated BEX2 promoted tumorigenia through STAT3. According to the TCGA database, PEG3 expression was positively correlated with BEX2 expression, and the investigators found that PEG3 silencing significantly reduced BEX2 expression at both protein and mRNA levels. These results suggest that mutant Kras and mTOR crosstalk drive HCC development through PEG3/STAT3/BEX2 signaling [107]. Daisuke Fukushi et al. found that BEX2 was highly expressed in HCC compared with adjacent normal lesions, and was located in Ki67 negative cancer cells in HCC tissue. They have additionally discovered that BEX2, while in a quiescent state within HCC, exerts an influence on the prognostic outcomes of individuals afflicted with liver cancer [108]. BEX2 maintains dormant tumor stem cells by inhibiting mitochondrial activity in cholangiocarcinoma cells [109]. In vivo experiments have shown that liver BEX1 expression increases during CDE diet-induced liver injury and is highly elevated mainly in LPC. BEX1^−/−^ Mice fed the CDE diet showed impaired LPC amplification and liver regeneration. BEX1 deficiency inhibited LPC proliferation and enhanced LPC apoptosis in vitro. At the same time, BEX1 deficiency inhibited LPCs colony formation, but had no effect on liver differentiation. At the same time, Peroxisome proliferator-activated receptor gamma (PPARG) has been detected in BEX1-deficient LPCs and mouse livers, and in terms of mechanism, BEX1 inhibits PPARG to promote LPC amplification [110]. The protein HBx is derived from the genome of hepatitis B virus (HBV) and serves as one of its encoded proteins [111]. Fuqiang Huang et al. discovered that the expression of BEX2 and osteopontin (OPN) was upregulated in hepatitis B transgenic mice and human HCC specimens. HBx is overexpressed in HepG2 cells, and ectopic expression of HBx significantly increases the protein levels of BEX2 and OPN. In addition, in liver cancer cells with high HBx expression, the absence of BEX2 effectively inhibits their tumorigenicity. These findings confirm the involvement of BEX2 in the pathogenesis of HCC and suggest that it may be a new therapeutic target against the invasion of HBV [112]. Lu Liu et al. showed that distinct biological processes were associated with the occurrence of sex-biased tumors in HCC. Moreover, it was noted that there exists an inverse relationship between the expression of BEX4 and the responsiveness of receptor tyrosine kinase inhibitor Lenvatinib. This suggests that targeting BEX4 could potentially augment the effectiveness of lenvatinib as an immune checkpoint inhibitor [113]. The mechanism by which liver diseases are associated with the BEX family proteins is shown in Fig. 7.

### BEX family proteins in heart disease

Heart failure (HF) is defined as a kind of myocardial disease, which evolves from the damage of myocardial function, mainly manifested as changes in hemodynamics, and is a clinical syndrome caused by various reasons [114]. Although drug treatment has reduced its associated incidence rate and mortality, it is still a treatment challenge for clinicians [115]. Researchers found that BEX1 is an inducible and unique pathogenic gene that depends on RNA complex in the process of HF and is harmful to the heart. It leads to disease progression during chronic pathological damage, and coordinates inflammatory signals through RNA dependent processing complex. In particular, the introduction of BEX1 greatly enhances the presence and durability of AU-rich elements, which encompass messenger RNAs that are frequently observed in genes associated with inflammation. In general, BEX1, as a mRNA dependent effector, enhances the gene expression promoting pathology in the process of HF [116]. Cardiomyositis is a cause of dilated cardiomyopathy [117]. It has various clinical manifestations. Once it is infected by cardiotropic virus, it will have an inadaptable viral post reaction, leading to dysfunction of myocardial cells and impairment of systolic function [118]. Previous research has provided evidence for the effectiveness of BEX1, as a novel stress-regulating proinflammatory factor in cardiac tissue, exerts a cardioprotective role during viral infection. Importantly, BEX1 exhibits broad-spectrum antiviral effects that are not solely reliant on the immune system. It maintains its antiviral efficacy in isolated cells and against diverse viral strains. In addition, the expression of BEX1 is controlled by interferon β, an antiviral protein, indicating that BEX1 could potentially have a significant impact on regulating the immune response against viral infections. Overall, BEX1 plays multifaceted roles in modulating the cardiac antiviral immune response [119].

### BEX family proteins in leukemia

**Fig. 8:**
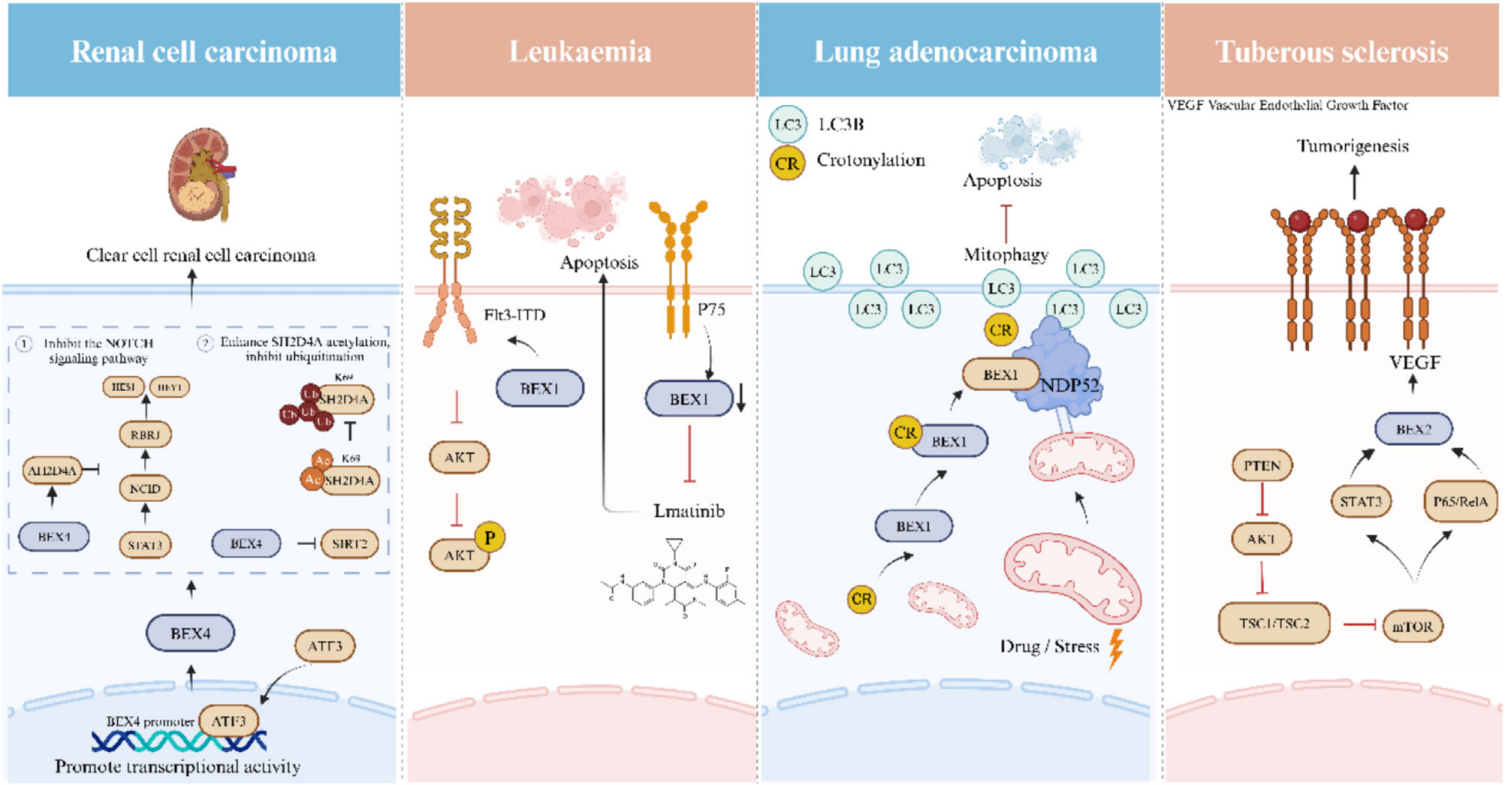
Mechanism diagram of BEX family proteins related to ccRCC, Leukemia, Lung adenocarcinoma, and Tuberous sclerosis.

The incidence of leukemia tends to be higher in highly developed parts of the world and among white Americans, and the time trends in incidence are dynamic and multifactorial [120,121]. Acute myeloid leukemia (AML) is a myeloid disorder that is the most common and most frequently studied leukemia [122]. Flt3 is a receptor tyrosine kinase expressed by immature hematopoietic cells, and mutations in the Fit3 gene are detected in approximately 30 % of AML [123]. By Affyphidogram HG-U133 A microarray (Santa Clara, CA, USA), BEX1 was expressed in 82 % of MLL mutant (MLLmu) AML cell lines and 18 % of MLL wild-type (MLLwt) AML cell lines. BEX1-positive MLLwt cell lines show very weak BEX1 signaling, supporting the idea of a positive correlation between MLL gene aberration and high BEX1 expression levels in AML. In summary, BEX1 is a candidate gene for the diagnosis of MLLmuAML [124]. The researchers performed disulfide bond transformation assays on MLLmu and MLLwt cell lines, showing a strong correlation between MLLmu, BEX1 expression and hypomethylation. With 5 % methylation in the MLLmu cell line, BEX2 is also a candidate gene for the diagnosis of MLLmuAML [125]. Existing studies have shown that BEX1 is a regulator of AML driven by a new tumor gene Flt3-ITD. BEX1 is localized in the cytoplasm, which can significantly reduce the phosphorylation of AKT induced by Flt3-ITD, but does not affect the phosphorylation of STAT5 and ERK1/2. The loss of BEX1 expression significantly enhances the carcinogenic signal and reduces the overall survival rate of patients [126]. In addition, Ding et al. conducted a study where they successfully established a K562 cell line resistant to imatinib. Upon analyzing the gene expression profile of these cells, it was discovered that BEX1 is regulated by the p75 neurotrophin receptor pathway in imatinib-resistant K562 cells. The results suggest that BEX1 may have a significant impact on inducing cell apoptosis in the process of drug resistance development [127]. The low methylation and high expression of BEX2 in MLLmu AML cell lines may be related to the expression of MLL fusion protein, while the high methylation and low expression of BEX2 in MLLwt AML cell lines may be regulated by epigenetic modification [128]. The mechanism of the BEX family proteins associated with leukemia is shown in Fig. 8.

### BEX family proteins in other diseases

In addition to the above diseases, BEX family proteins also play a very important role in other diseases. BEX4 enhances the expression of SH2D4A (NOTCH pathway inhibitor) by inhibiting SIRT2 activity, thereby promoting its tumor suppressive effect in inhibiting the progression of renal clear cell carcinoma (ccRCC). Meanwhile, the transcriptional activity of BEX4 is positively regulated by ATF3, suggesting that BEX4 may be a potential diagnostic and therapeutic target for ccRCC [129]. Ning Mu et al. found that BEX2 is overexpressed in LAC. Crotonylation of BEX2 enhances the interaction between LC3B and NDP52, thereby regulating mitochondrial autophagy. Among them, the K59R mutation of BEX2 inhibits mitochondrial autophagy by affecting the interaction of NDP52 and LC3B. At the same time, the expression of BEX2 increased after anti-cancer drug treatment, and BEX2 promoted tumor growth and inhibited apoptosis by regulating mitochondrial autophagy in vivo [130]. The reasons for Esophageal squamous cell carcinoma (ESCC) are many and vary from region to region. High incidence areas include East Asia to Central Asia, East Africa along the East African Rift Valley, and South Africa [131]. The histological subtype of esophageal carcinoma known as ESCC holds paramount importance among various subtypes within this category [132]. Geng et al showed in vitro that BEX1 overexpression inhibited ESCC cell colony formation and proliferation, and in vivo tumorigenesis assay showed that BEX1 overexpression inhibited ESCC tumor growth in mice. Mechanistically, up-regulation of BEX1 leads to decreased expression and phosphorylation of NF-κB p65, thereby inhibiting the NF-κB signaling pathway [3]. BEX2 is a novel downstream effector of mTOR, a mechanotarget of rapamycin, whose expression is regulated by the mTOR inhibitor rapamycin. BEX2 is associated with activation of mTOR signaling pathway, tumorigenesis and angiogenesis, and may be a potential target for the treatment of tumors associated with abnormal activation of mTOR signaling pathway [18]. The mechanisms of the BEX family proteins associated with ccRCC, leukemia, lung adenocarcinoma, and tuberous sclerosis are shown in Fig. 8.

## Therapeutic potential of targeting BEX family proteins in human diseases

In recent years, cancer and many other diseases have remained a global public health challenge, and although researchers and physicians have invested a great deal of time and effort in research and treatment, the complexity and heterogeneity of these diseases make it extremely difficult to overcome them. Therefore, it is the desire of the whole society to reduce the burden of disease, increase the healthy life expectancy of residents, and ensure people's health in an all-round and whole-cycle manner. In view of the important function of the BEX family proteins, this paper collected the current research on diseases related to the BEX family proteins. The commonly used animal modeling methods and time are shown in Table 1. The BEX family proteins is expected to become a potential therapeutic target for related diseases.

**Table 1:** Animal modeling models and modeling cycles for BEX family proteins related diseases.

BEX member	Related diseases	Experimental animal model /cell model	Cell/animal modeling cycle	Reference
BEX	Retinal	Neurostroke model	28 days	[58]
BEX1	Liver cancer	A HCC mode l of BEX1^-/-^ mice	7 weeks	[37]
	Cardiomyopathy	A neuroendocrine model of cardiac injury	4 weeks	[116]
	Intracerebral hemorrhage	Rat intracerebral hemorrhage model	6 h-14 days	[59]
	Viral myocarditis	Using a murine model of CVB3-induced viral myocarditis	28 days	[119]
	Glioblastoma multiforme	U87MG xenograft GBM mouse model	23 days	[88]
	Axonal regeneration	Pmp22-KO mice and mice over-expressing Pmp22	28 days	[17]
	Ciliogenesis	Production of BEX1 mutant mouse strains using CRISPR-Cas9 technology	/	[31]
	Chronic liver disease	CDE diet-induced liver injury model	3 weeks	[110]
	Acute myeloid leukemia	Flt3-ITD-induced tumor model in mice	24 days	[126]
	Skeletal muscle regeneration	BEX1 knock out mice.	/	[40]
	Glioma	Inject glioma cells stably expressing LINC00526 into nude mice	28 days	[86]
	Esophageal squamous cell cancer	Xenograft tumor assay	/	[3]
	Oral squamous cell carcinoma	In vivo tumorigenic model of SCID mice	/	[73]
	Non-alcoholic steatohepatitis	Cx32ΔTg rats	12 weeks	[133]
	Hepatocellular carcinoma	B6C3F1 mice treated with diethylnitrosamine and carbon tetrachloride to establish a late stage liver fibrosis model	9 or 14 weeks	[102]
	Hepatoblastoma	Nude mice xenograft model	40 days	[103]
	Hepatocellular carcinoma	Sorafenib-resistant HCC xenograft model	least 2 weeks	[104]
BEX2	Hepatocellular carcinoma	NOG mice in vivo tumor model	8 weeks	[108]
	Intrahepatic cholangiocarcinoma	Patient derived xenograft (PDX) cell NOG mice in vivo tumor model	12 weeks	[109]
	Colorectal cancer	Subcutaneous xenograft model	5 weeks	[81]
	Colorectal cancer	The orthotopic mouse model of CRC	45 days	[82]
	Hepatocellular carcinoma	A nude mouse xenograft tumor model	35 days	[106]
	Tumor	Subcutaneous tumor model in nude mice	/	[18]
	Hepatocellular carcinoma	MHCC97H cell xenotransplantation Immunocompromised nude mice(BALB/c) ; HBV transgenic mice	/	[112]
	Gliomas	Hs683 cells were transplanted into nude mice brains.	/	[87]
	Malignant Glioma	U87 glioma cells Xenograft NCR(nu/nu) mice	28 days	[93]
	Stem/progenitor cells	BEX2-EGFP knock-in–knock-out mice	9 weeks	[66]
	Alcoholic hepatitis	EtOH	10 days	[105]
	Hepatocellular carcinoma	Liver-specific Cre recombinase mouse line	280 days	[107]
BEX3	Nasopharyngeal carcinoma	Resistant HONE1 cells Xenotransplantation athymic nu/nu mice	28 days	[76]
BEX4	Microtubule acetylation	Subcutaneous injection of retrovirus mediated BALB/3T3 cells into mice	7 weeks	[51]
	Oral squamous cell carcinoma	Athymic nu/nu mice	30 days	[4]
	Testicular dysfunction	A male reproductive dysfunction model	5 days	[29]

Animal experiments play a crucial role in pharmacological research, they can simulate the characteristics and pathophysiological processes of target diseases, help scientists observe the development of diseases, and test the efficacy and safety of drugs. The similarity between animals and humans is particularly important in disease research because it allows researchers to replicate human disease in animals, allowing for more accurate observation of the model's experimental results and comparative studies with human disease. In particular, the BEX family proteins plays an important role in a variety of biological processes, including inhibiting the proliferation and metastasis of cancer cells, and participating in various biological processes such as cell differentiation, cell proliferation, and cell apoptosis. Therefore, BEX family proteins related disease research is of great significance in animal experiments, they not only help to reveal the molecular mechanism of the disease, but also may become an important target or biomarker for the development of new drugs. The study of the function and mechanism of BEX family proteins through animal models can provide new strategies and methods for the treatment of related diseases.

Taken together, the above studies suggest that inhibition or activation of the BEX family proteins has considerable potential in the therapeutic management of a variety of diseases, including cancer. Given the effectiveness of the BEX family proteins as a therapeutic target, the BEX family proteins is expected to be a viable therapeutic candidate for improving the disease and deserves further study.

## Discussion and potential directions

A large body of evidence suggests that the BEX family proteins plays a key role in the development of disease and cancer. Therefore, the BEX family proteins has become an important therapeutic target. Jorma J. de Ronde et al. identified the markers of drug resistance by detecting unbalanced differential signals (DIDS). The analysis results showed that five genes, including BEX1, SERPINA6, AGTR1, may lead to a better understanding of the clinically observed mechanism of chemotherapy resistance, and may help to identify breast cancer [134]. The potential contribution of the BEX family to the progression of LAD implies their potential utility as distinctive and highly responsive indicators for the diagnosis of LUAD. Notably, BEX4 shows promise as a prognostic indicator for both early and late stages of LUAD, making it a potential candidate for tumor marker identification [135].

The BEX family proteins still has many unsolved mysteries and potential research directions in disease research. First, the expression of BEX family genes varies significantly across multiple cancers and is associated with overall survival (OS) in patients, suggesting that they may be important prognostic predictors. However, the role of BEX family members in tumor cells is not completely clear, and even the conclusions of existing studies are divergent. Some studies have suggested that this family of proteins may promote tumor cell proliferation, while others have found that it has an inhibitory effect. This functional difference may be related to the following factors: the complexity of tumor pathogenesis, the heterogeneity of molecular characteristics and signaling pathways in different tumors, which may lead to the different roles of BEX proteins; The function of BEX proteins may depend on the cellular background of a specific tumor, microenvironmental conditions (such as immune regulation, metabolic status), and the characteristics of cancer stem cells. At present, the research on BEX family proteins is still in the preliminary stage, and some conclusions are contradictory, so more experimental evidence is needed to clarify the regulatory mechanism. In addition, the expression of BEX family genes is related to the tumor microenvironment and tumor stem cells, which provides a new direction for regulating the expression of BEX family genes to reshape the tumor microenvironment and affect the proliferation of tumor stem cells.

In summary, our current understanding suggests that BEX family members play different functions in different diseases. However, there are still some unanswered questions, especially for less studied BEX family members such as BEX4 and BEX5. Future studies should focus on exploring the links between these BEX family proteins and various diseases and cancers to better assess their biological role in humans. To achieve this goal, the adoption of specific experimental models will be crucial. For example, CRISPR knockout models in relevant cell lines or animal models will enable precise deletion of BEX genes, allowing detailed study of their essential functions in cellular processes such as proliferation, differentiation, and apoptosis. In addition, the use of patient-derived organoids could provide a more physiologically relevant system to study the role of BEX family proteins in disease, especially to understand their contribution to tumorigenesis and drug resistance. By combining these cutting-edge experimental approaches, we can gain a more complete understanding of the multifaceted roles of BEX family proteins and may identify novel therapeutic targets. In the future, the search for links between BEX family proteins and disease and cancer will help to assess the biological role of members of this protein family in humans.

Compliance with Ethics Requirements

This research did not require IRB (institutional review board) approval because this is a review article.

## CRediT authorship contribution statement

**Pingping Wang:** Writing-original draft. **Ziyan Chen:** Sort out the data. **Hongyan Zhang:** Validation. **Yandan Lu:** Formal analysis. **Licheng Zhou:** Collating of data. **Chenghang Gong:** Collating of data. **Dongyang An:** Conceptualization. **Xianan Sang:** Methodology. **Kuilong Wang:** Data curation. **Min Hao:** Writing – review & editing. **Gang Cao:** Supervision.

## Declaration of competing interest

The authors declare that they have no known competing financial interests or personal relationships that could have appeared to influence the work reported in this paper.

## References

[1] Yi M., Li T., Niu M., Zhang H., Wu Y., Wu K. (2024). Targeting cytokine and chemokine signaling pathways for cancer therapy. Signal Transduct. Target. Ther..

[2] Fernandez E.M., Diaz-Ceso M.D., Vilar M. (2015). Brain expressed and X-linked (Bex) proteins are intrinsically disordered proteins (IDPs) and form new signaling hubs. PLoS One.

[3] Geng H.T., Cheng Z.W., Cao R.J., Wang Z.B., Xing S.Z., Guo C. (2018). Low expression of BEX1 predicts poor prognosis in patients with esophageal squamous cell cancer. Oncol. Rep..

[4] Gao W., Li J.-Z.-H., Chen S.-Q., Chu C.-Y., Chan J.-Y.-W., Wong T.-S. (2016). Decreased brain-expressed X-linked 4 (BEX4) expression promotes growth of oral squamous cell carcinoma. J. Exp. Clin. Cancer Res..

[5] Naderi A, Liu J, Francis GD (2012). A feedback loop between BEX2 and ErbB2 mediated by c-Jun signaling in breast cancer. Int J Cancer.

[6] Winter E.E., Mammalian P.CP. (2005). Bex, WEX and GASP genes: coding and non-coding chimaerism sustained by gene conversion events. BMC Evol. Biol..

[7] Alvarez E., Zhou W., Witta S.E., Freed C.R. (2005). Characterization of the Bex gene family in humans, mice, and rats. Gene.

[8] Zhang L. (2008). Adaptive evolution and frequent gene conversion in the brain expressed X-linked gene family in mammals. Biochem. Genet..

[9] Zhou X., Sumrow L., Tashiro K., Sutherland L., Liu D., Qin T. (2022). Mutations linked to neurological disease enhance self-association of low-complexity protein sequences. Science.

[10] Permyakov E.A., Fernandez E.M., Díaz-Ceso M.D., Vilar M. (2015). Brain expressed and X-Linked (Bex) proteins are intrinsically disordered proteins (IDPs) and form new signaling hubs. PLoS One.

[11] Faria TN, LaRosa GJ, Wilen E, Liao J, Gudas LJ (1998). Characterization of genes which exhibit reduced expression during the retinoic acid-induced differentiation of F9 teratocarcinoma cells: involvement of cyclin D3 in RA-mediated growth arrest. Mol Cell Endocrinol.

[12] Brown AL, Kay GF (1999). Bex1, a gene with increased expression in parthenogenetic embryos, is a member of a novel gene family on the mouse X chromosome. Hum Mol Genet.

[13] Williams J.W., Hawes S.M., Patel B., Latham K.E. (2002). Trophectoderm-specific expression of the X-linkedBex1/Rex3 gene in preimplantation stage mouse embryos. Mol. Reprod. Dev.

[14] Yang QS, Xia F, Gu SH, Yuan HL, Chen JZ, Yang QS (2002). Cloning and expression pattern of a spermatogenesis-related gene, BEX1, mapped to chromosome Xq22. Biochem Genet.

[15] Yan Z., Choi S., Liu X., Zhang M., Schageman J.J., Lee S.Y. (2003). Highly coordinated gene regulation in mouse skeletal muscle regeneration. J. Biol. Chem..

[16] Vilar M., Murillo-Carretero M., Mira H., Magnusson K., Besset V., Ibáñez C.F. (2006). Bex1, a novel interactor of the p75 neurotrophin receptor, links neurotrophin signaling to the cell cycle. EMBO J..

[17] Khazaei M.R., Halfter H., Karimzadeh F., Koo J.H., Margolis F.L., Young P. (2010). Bex1 is involved in the regeneration of axons after injury. J. Neurochem..

[18] Hu Z., Wang Y., Huang F., Chen R., Li C., Wang F. (2015). Brain-expressed X-linked 2 is pivotal for hyperactive mechanistic target of rapamycin (mTOR)-mediated tumorigenesis. J. Biol. Chem..

[19] Yamamoto-Shimojima K., Osawa M., Saito M.K., Yamamoto T. (2020). iPSCs established from a female patient with Xq22 deletion confirm that BEX2 escapes from X-chromosome inactivation. Congenit. Anom..

[20] Han C. (2005). Human Bex2 interacts with LMO2 and regulates the transcriptional activity of a novel DNA-binding complex. Nucleic Acids Res..

[21] Bahadar N, Ullah H, Adlat S, Kumar Sah R, Zun Zaw Myint M, Mar Oo Z (2021). Analyzing differentially expressed genes and pathways of Bex2-deficient mouse lung via RNA-Seq. Turk J Biol.

[22] Rapp JF G., Klaudiny J., Mucha J., Wempe F., Zimmer M., Scheit K.H. (1990). Characterization of three abundant mRNAs from human ovarian granulosa cells. DNA Cell Biol..

[23] Hajime Sano M.J.M., Monoo K., Close MT-AS L.G. (2001). Expression of p75NTR and its associated protein NADE in the rat cochlea. Laryngoscope.

[24] Kim A.-J., Lee C.-S., Schlessinger D. (2004). Bex3 associates with replicating mitochondria and is involved in possible growth control of F9 teratocarcinoma cells. Gene.

[25] Silman I., Cabral K.M.S., Raymundo D.P., Silva V.S., Sampaio L.A.G., Johanson L. (2015). Biophysical studies on BEX3, the p75NTR-associated cell death executor, reveal a high-order oligomer with partially folded regions. PLoS One.

[26] do Amaral MJ., Araujo TS., Diaz NC., Accornero F., Polycarpo CR., Cordeiro Y. (2020). Phase separation and disorder-to-order transition of human brain expressed X-linked 3 (hBEX3) in the presence of small fragments of tRNA. J. Mol. Biol..

[27] Mukai J., Hachiya T., Shoji-Hoshino S., Kimura M.T., Nadano D., Suvanto P. (2000). NADE, a p75NTR-associated cell death executor, is involved in signal transduction mediated by the common neurotrophin receptor p75NTR. J. Biol. Chem..

[28] Parkhurst C.N., Zampieri N., Chao M.V. (2010). Nuclear localization of the p75 neurotrophin receptor intracellular domain. J. Biol. Chem..

[29] Yu W., Yaping L., Mingjun W., Jie H., Xiaogang L., Gang L. (2017). BEX4 upregulation alters Sertoli cell growth properties and protein expression profiles: an explanation for cadmium-induced testicular Sertoli cell injury. J. Biochem. Mol. Toxicol..

[30] van der Lee R., Buljan M., Lang B., Weatheritt R.J., Daughdrill G.W., Dunker A.K. (2014). Classification of intrinsically disordered regions and proteins. Chem. Rev..

[31] Hibino E., Ichiyama Y., Tsukamura A., Senju Y., Morimune T., Ohji M. (2022). Bex1 is essential for ciliogenesis and harbours biomolecular condensate-forming capacity. BMC Biol..

[32] Keller A., Margolis F.L. (1975). Immunological studies of the rat olfactory marker protein. J. Neurochem..

[33] Koo J.H., Saraswati M., Margolis F.L. (2005). Immunolocalization of Bex protein in the mouse brain and olfactory system. J Comp Neurol.

[34] Koo J.H., Gill S., Pannell L.K., Menco B.P.M., Margolis J.W., Margolis F.L. (2004). The interaction of Bex and OMP reveals a dimer of OMP with a short half-life. J. Neurochem..

[35] Behrens M., Margolis J.W., Margolis F.L. (2003). Identification of members of the Bex gene family as olfactory marker protein (OMP) binding partners. J. Neurochem..

[36] Chuang L.S.H., Ito Y. (2010). RUNX3 is multifunctional in carcinogenesis of multiple solid tumors. Oncogene.

[37] Wang Q., Liang N., Yang T., Li Y., Li J., Huang Q. (2021). DNMT1-mediated methylation of BEX1 regulates stemness and tumorigenicity in liver cancer. J. Hepatol..

[38] Radha G., Raghavan S.C. (2017). BCL2: a promising cancer therapeutic target. Biochim. Biophys. Acta.

[39] Villunger A., Xiao Q., Hu Y., Liu Y., Wang Z., Geng H. (2014). BEX1 promotes imatinib-induced apoptosis by binding to and antagonizing BCL-2. PLoS One.

[40] Koo J.H., Smiley M.A., Lovering R.M., Margolis F.L. (2007). Bex1 knock out mice show altered skeletal muscle regeneration. Biochem. Biophys. Res. Commun..

[41] Han Q.-Y., Fan Y.-H., Wang Y.-L., Zhang S.-D., Han C.-Y. (2012). BEX2 regulates cell cycle through the interaction with INI1/hSNF5. Hereditas (Beijing).

[42] Naderi A, Liu J, Hughes-Davies L (2010). BEX2 has a functional interplay with c-Jun/JNK and p65/RelA in breast cancer. Mol Cancer.

[43] Iliaki S., Beyaert R., Afonina I.S. (2021). Polo-like kinase 1 (PLK1) signaling in cancer and beyond. Biochem. Pharmacol..

[44] Lee J.-K., Ha G.-H., Kim H.-S., Lee C.-W. (2018). Oncogenic potential of BEX4 is conferred by Polo-like kinase 1-mediated phosphorylation. Exp. Mol. Med..

[45] Hotchkiss R.S., Strasser A., McDunn J.E., Swanson P.E. (2009). Cell Death. N. Engl. J. Med..

[46] Sidhar H., Giri R.K. (2017). Induction of Bex genes by curcumin is associated with apoptosis and activation of p53 in N2a neuroblastoma cells. Sci. Rep..

[47] Judd J., Lovas J., Huang G.N. (2019). Defined factors to reactivate cell cycle activity in adult mouse cardiomyocytes. Sci. Rep..

[48] Stockwell B.R., Jiang X., Gu W. (2020). Emerging mechanisms and disease relevance of ferroptosis. Trends Cell Biol..

[49] Yang Y., Tetti M., Vohra T., Adolf C., Seissler J., Hristov M. (2021). BEX1 is differentially expressed in aldosterone-producing adenomas and protects human adrenocortical cells from ferroptosis. Hypertension.

[50] Jeong Ae Park J.-Y.-L., Sato T.-A., Koh J.-Y. (2000). Co-induction of p75NTR and p75NTR-associated death executor in neurons after zinc exposure in cortical culture or transient ischemia in the rat. J. Neurosci..

[51] Lee J.-K., Lee J., Go H., Lee C.G., Kim S., Kim H.-S. (2016). Oncogenic microtubule hyperacetylation through BEX4-mediated sirtuin 2 inhibition. Cell Death Dis..

[52] Doi T., Ogawa H., Tanaka Y., Hayashi Y., Maniwa Y. (2020). Bex1 significantly contributes to the proliferation and invasiveness of malignant tumor cells. Oncol. Lett..

[53] Karakoula K., Jacques T.S., Phipps K.P., Harkness W., Thompson D., Harding B.N. (2014). Epigenetic genome-wide analysis identifies BEX1 as a candidate tumour suppressor gene in paediatric intracranial ependymoma. Cancer Lett..

[54] Batlle E., Clevers H. (2017). Cancer stem cells revisited. Nat. Med..

[55] Nassar D., Blanpain C. (2016). Cancer stem cells: basic concepts and therapeutic implications. Annu. Rev. Pathol..

[56] Saijoh S., Nakamura-Shima M., Shibuya-Takahashi R., Ito R., Sugawara A., Yamazaki T. (2021). Discovery of a chemical compound that suppresses expression of BEX2, a dormant cancer stem cell-related protein. Biochem. Biophys. Res. Commun..

[57] Zhao Z., Li J., Tan F., Gao S., He J. (2018). mTOR up-regulation of BEX4 promotes lung adenocarcinoma cell proliferation by potentiating OCT4. Biochem. Biophys. Res. Commun..

[58] Bernstein S.L., Jae Hyung Koo B.J.S., Guo Y., Margolis F.L. (2016). Analysis of optic nerve stroke by retinal Bex expression. Mol. Vis..

[59] He M., Wang Y., Shen J., Duan C., Lu X., Li J. (2018). Bex1 attenuates neuronal apoptosis in rat intracerebral hemorrhage model. Pathology - Research and Practice.

[60] Li P., Ma K., Wu H.-Y., Wu Y.-P., Li B.-X. (2017). Isoflavones Induce BEX2-dependent autophagy to prevent ATR-induced neurotoxicity in SH-SY5Y Cells. Cell. Physiol. Biochem..

[61] Li P., Yao L.-Y., Jiang Y.-J., Wang D.-D., Wang T., Wu Y.-P. (2021). Soybean isoflavones protect SH-SY5Y neurons from atrazine-induced toxicity by activating mitophagy through stimulation of the BEX2/BNIP3/NIX pathway. Ecotoxicol. Environ. Saf..

[62] Calvo L., Anta B., López-Benito S., Martín-Rodriguez C., Lee F.S., Pérez P. (2015). Bex3 dimerization regulates NGF-dependent neuronal survival and differentiation by EnhancingtrkAGene transcription. J. Neurosci..

[63] Yasui S., Tsuzaki K., Ninomiya H., Floricel F., Asano Y., Maki H. (2007). The TSC1 gene product hamartin interacts with NADE. Mol. Cell. Neurosci..

[64] Navas-Pérez E., Vicente-García C., Mirra S., Burguera D., Fernàndez-Castillo N., Ferrán J.L. (2020). Characterization of an eutherian gene cluster generated after transposon domestication identifies Bex3 as relevant for advanced neurological functions. Genome Biol..

[65] Roskams TA, Libbrecht L, Desmet VJ (2003). Progenitor cells in diseased human liver. Semin Liver Dis.

[66] Ito K., Yamazaki S., Yamamoto R., Tajima Y., Yanagida A., Kobayashi T. (2014). Gene targeting study reveals unexpected expression of brain-expressed X-linked 2 in endocrine and tissue stem/progenitor cells in mice. J. Biol. Chem..

[67] Jiang C., Wang J.-H., Yue F., Kuang S. (2016). The brain expressed x-linked gene 1 (Bex1) regulates myoblast fusion. Dev. Biol..

[68] Guan W.-L., He Y., Xu R.-H. (2023). Gastric cancer treatment: recent progress and future perspectives. J. Hematol. Oncol..

[69] Smyth E.C., Nilsson M., Grabsch H.I., van Grieken N.C., Lordick F. (2020). Gastric cancer. Lancet.

[70] Yasumoto A., Fujimori H., Mochizuki M., Shibuya-Takahashi R., Nakamura-Shima M., Shindo N. (2023). BEX2 is poor prognostic factor and required for cancer stemness in gastric cancer. Biochem. Biophys. Res. Commun..

[71] Zhu C., Xiao D. (2020). Aberrant brain-expressed X-linked 4 (BEX4) expression is a novel prognostic biomarker in gastric cancer. Medicine.

[72] Tan Y., Wang Z., Xu M., Li B., Huang Z., Qin S. (2023). Oral squamous cell carcinomas: state of the field and emerging directions. Int. J. Oral Sci..

[73] Lee C.H., Wong T.S., Chan J.Y., Lu S.C., Lin P., Cheng A.J. (2013). Epigenetic regulation of the X-linked tumour suppressors BEX1 and LDOC1 in oral squamous cell carcinoma. J. Pathol..

[74] Chen Y.P., Chan A.T.C., Le Q.T., Blanchard P., Sun Y., Ma J. (2019). Nasopharyngeal carcinoma. Lancet.

[75] Chen W., Zheng R., Baade P.D., Zhang S., Zeng H., Bray F. (2016). Cancer statistics in China, 2015. CA Cancer J. Clin..

[76] Gao W., Li J.-Z.-H., Chen S.-Q., Chu C.-Y., Chan J.-Y.-W., Wong T.-S. (2017). BEX3 contributes to cisplatin chemoresistance in nasopharyngeal carcinoma. Cancer Med..

[77] Shin A.E., Giancotti F.G., Rustgi A.K. (2023). Metastatic colorectal cancer: mechanisms and emerging therapeutics. Trends Pharmacol. Sci..

[78] Dekker E., Tanis P.J., Vleugels J.L.A., Kasi P.M., Wallace M.B. (2019). Colorectal cancer. Lancet.

[79] Naderi A. (2019). Molecular functions of brain expressed X-linked 2 (BEX2) in malignancies. Exp. Cell Res..

[80] Liu F., Huang W., Hong J., Cai C., Zhang W., Zhang J. (2020). Long noncoding RNA LINC00630 promotes radio-resistance by regulating BEX1 gene methylation in colorectal cancer cells. IUBMB Life.

[81] Hu Y., Xiao Q., Chen H., He J., Tan Y., Liu Y. (2017). BEX2 promotes tumor proliferation in colorectal cancer. Int. J. Biol. Sci..

[82] Tan Y., Hu Y., Xiao Q., Tang Y., Chen H., He J. (2020). Silencing of brain-expressed X-linked 2 (BEX2) promotes colorectal cancer metastasis through the Hedgehog signaling pathway. Int. J. Biol. Sci..

[83] Verdugo E., Puerto I., Medina M.Á. (2022). An update on the molecular biology of glioblastoma, with clinical implications and progress in its treatment. Cancer Commun..

[84] Carlsson S.K., Brothers S.P., Wahlestedt C. (2014). Emerging treatment strategies for glioblastoma multiforme. EMBO Mol. Med..

[85] Uddin M.S., Mamun A.A., Alghamdi B.S., Tewari D., Jeandet P., Sarwar M.S. (2022). Epigenetics of glioblastoma multiforme: from molecular mechanisms to therapeutic approaches. Semin. Cancer Biol..

[86] Yan J., Li Y., Xu C., Tang B., Xie S., Hong T. (2021). Long noncoding RNA LINC00526 represses glioma progression via regulating miR-5581-3p/BEX1. J. Oncol..

[87] Mercier M.L., Fortin S., Mathieu V., Roland I., Spiegl-Kreinecker S., Haibe-Kains B. (2009). Galectin 1 proangiogenic and promigratory effects in the Hs683 oligodendroglioma model are partly mediated through the control of BEX2 expression. Neoplasia.

[88] Lee S., Kang H., Shin E., Jeon J., Youn H., Youn B. (2021). BEX1 and BEX4 induce GBM progression through regulation of actin polymerization and activation of YAP/TAZ signaling. Int. J. Mol. Sci..

[89] Zhou X., Xu X., Meng Q., Hu J., Zhi T., Shi Q. (2012). Bex2 is critical for migration and invasion in malignant glioma cells. J. Mol. Neurosci..

[90] Zhou X., Meng Q., Xu X., Zhi T., Shi Q., Wang Y. (2012). Bex2 regulates cell proliferation and apoptosis in malignant glioma cells via the c-Jun NH2-terminal kinase pathway. Biochem. Biophys. Res. Commun..

[91] Meng Q., Zhi T., Chao Y., Nie E., Xu X., Shi Q. (2014). Bex2 controls proliferation of human glioblastoma cells through NF-κB signaling pathway. J. Mol. Neurosci..

[92] Nie E., Zhang X., Xie S., Shi Q., Hu J., Meng Q. (2015). β-Catenin is involved in Bex2 down-regulation induced glioma cell invasion/migration inhibition. Biochem. Biophys. Res. Commun..

[93] Foltz G., Ryu G.-Y., Yoon J.-G., Nelson T., Fahey J., Frakes A. (2006). Genome-wide analysis of epigenetic silencing identifies BEX1 and BEX2 as candidate tumor suppressor genes in malignant glioma. Cancer Res..

[94] Aisa A., Tan Y., Li X., Zhang D., Shi Y., Yuan Y. (2022). Comprehensive analysis of the brain-expressed X-Link protein family in glioblastoma multiforme. Front. Oncol..

[95] Nolan E., Lindeman G.J., Visvader J.E. (2023). Deciphering breast cancer: from biology to the clinic. Cell.

[96] Harbeck N., Gnant M. (2017). Breast cancer. Lancet.

[97] Xu X., Zhang M., Xu F., Jiang S. (2020). Wnt signaling in breast cancer: biological mechanisms, challenges and opportunities. Mol. Cancer.

[98] Naderi A., Teschendorff A.E., Beigel J., Cariati M., Ellis I.O., Brenton J.D. (2007). BEX2 is overexpressed in a subset of primary breast cancers and mediates nerve growth factor/nuclear factor-κB inhibition of apoptosis in breast cancer cell lines. Cancer Res..

[99] Naderi A., Liu J., Bennett I.C. (2009). BEX2 regulates mitochondrial apoptosis and G1 cell cycle in breast cancer. Int. J. Cancer.

[100] Miyajima A., Tanaka M., Itoh T. (2014). Stem/progenitor cells in liver development, homeostasis, regeneration, and reprogramming. Cell Stem Cell.

[101] Bruix J., Han K.H., Gores G., Llovet J.M., Mazzaferro V. (2015). Liver cancer: approaching a personalized care. J. Hepatol..

[102] Uehara T, Ainslie GR, Kutanzi K, Pogribny IP, Muskhelishvili L, Izawa T (2013). Molecular mechanisms of fibrosis-associated promotion of liver carcinogenesis. Toxicol Sci.

[103] Wang Q., Liang N., Liu C., Li J., Bai Y., Lei S. (2023). BEX1 supports the stemness of hepatoblastoma by facilitating Warburg effect in a PPARγ/PDK1 dependent manner. Br. J. Cancer.

[104] Zhuang N., Gu Z., Feng J., Chai Z., Shan J., Qian C. (2023). BEX1 mediates sorafenib resistance in hepatocellular carcinoma by regulating AKT signaling. Cell. Signal..

[105] Zhou S., Zhong H., Wang Y., Wang X., Pan H., Liu X. (2023). JNK/MAPK pathway regulation by BEX2 gene silencing in alcoholic hepatitis mice. effects on oxidative stress. Alcohol: Clinical and Experimental Research.

[106] Wang X., Zhu W., Xu C., Wang F., Zhu X., Sun Y. (2019). MicroRNA-370 functions as a tumor suppressor in hepatocellular carcinoma via inhibition of the MAPK/JNK signaling pathway by targeting BEX2. J. Hum. Genet..

[107] Luo Y.-D., Liu X.-Y., Fang L., Yu H.-Q., Zhang Y.-J., Chen M. (2022). Mutant Kras and mTOR crosstalk drives hepatocellular carcinoma development via PEG3/STAT3/BEX2 signaling. Theranostics.

[108] Fukushi D., Shibuya-Takahashi R., Mochizuki M., Fujimori H., Kogure T., Sugai T. (2021). BEX2 is required for maintaining dormant cancer stem cell in hepatocellular carcinoma. Cancer Sci..

[109] Tamai K., Nakamura-Shima M., Shibuya-Takahashi R., Kanno S.-I., Yasui A., Mochizuki M. (2020). BEX2 suppresses mitochondrial activity and is required for dormant cancer stem cell maintenance in intrahepatic cholangiocarcinoma. Sci. Rep..

[110] Gu Y., Wei W., Cheng Y., Wan B., Ding X., Wang H. (2018). A pivotal role of BEX1 in liver progenitor cell expansion in mice. Stem Cell Res Ther.

[111] Pollicino T., Saitta C., Raimondo G. (2011). Hepatocellular carcinoma: the point of view of the hepatitis B virus. Carcinogenesis.

[112] Huang F, Cai P, Wang Y, Zhou X, Chen H, Liao W (2017). Up-regulation of brain-expressed X-linked 2 is critical for hepatitis B virus X protein-induced hepatocellular carcinoma development. Oncotarget.

[113] Liu L., Yu K., Huang C., Huo M., Li X., Yin R. (2022). Sex differences in hepatocellular carcinoma indicated BEX4 as a potential target to improve efficacy of lenvatinib plus immune checkpoint inhibitors. J. Cancer.

[114] Savarese G., Becher P.M., Lund L.H., Seferovic P., Rosano G.M.C., Coats A.J.S. (2022). Global burden of heart failure: a comprehensive and updated review of epidemiology. Cardiovasc. Res..

[115] Khan M.S., Arshad M.S., Greene S.J., Van Spall H.G.C., Pandey A., Vemulapalli S. (2023). Artificial intelligence and heart failure: a state‐of‐the‐art review. Eur. J. Heart Fail..

[116] Accornero F., Schips T.G., Petrosino J.M., Gu S.-Q., Kanisicak O., van Berlo J.H. (2017). BEX1 is an RNA-dependent mediator of cardiomyopathy. Nat. Commun..

[117] Sagar S., Liu P.P., Cooper L.T. (2012). Myocarditis Lancet.

[118] Pollack A., Kontorovich A.R., Fuster V., Dec G.W. (2015). Viral myocarditis–diagnosis, treatment options, and current controversies. Nat. Rev. Cardiol..

[119] Belov G.A., Martens C.R., Dorn L.E., Kenney A.D., Bansal S.S., Yount J.S. (2022). BEX1 is a critical determinant of viral myocarditis. PLoS Pathog..

[120] Bispo J.A.B., Pinheiro P.S., Kobetz E.K. (2020). Epidemiology and Etiology of Leukemia and Lymphoma. Cold Spring Harb. Perspect. Med..

[121] Greaves M. (2016). Leukaemia 'firsts' in cancer research and treatment. Nat. Rev. Cancer.

[122] Whiteley A.E., Price T.T., Cantelli G., Sipkins D.A. (2021). Leukaemia: a model metastatic disease. Nat. Rev. Cancer.

[123] Gilliland D.G., Griffin J.D. (2002). The roles of FLT3 in hematopoiesis and leukemia. Blood.

[124] Quentmeier H., Tonelli R., Geffers R., Pession A., Uphoff C.C., Drexler H.G. (2005). Expression of BEX1 in acute myeloid leukemia with MLL rearrangements. Leukemia.

[125] Fischer C., Drexler H.G., Reinhardt J., Zaborski M., Quentmeier H. (2007). Epigenetic regulation of brain expressed X-linked-2, a marker for acute myeloid leukemia with mixed lineage leukemia rearrangements. Leukemia.

[126] Lindblad O, Li T, Su X, Sun J, Kabir NN, Levander F (2015). BEX1 acts as a tumor suppressor in acute myeloid leukemia. Oncotarget.

[127] Ding K., Su Y., Pang L., Lu Q., Wang Z., Zhang S. (2009). Inhibition of apoptosis by downregulation of hBex1, a novel mechanism, contributes to the chemoresistance of Bcr/Abl+ leukemic cells. Carcinogenesis.

[128] Röhrs S., Dirks W.G., Meyer C., Marschalek R., Scherr M., Slany R. (2009). Hypomethylation and expression of BEX2, IGSF4 and TIMP3 indicative of MLL translocations in Acute Myeloid Leukemia. Mol. Cancer.

[129] Li Z., Xin S., Huang L., Tian Y., Chen W., Liu X. (2024). BEX4 inhibits the progression of clear cell renal cell carcinoma by stabilizing SH2D4A, which is achieved by blocking SIRT2 activity. Oncogene.

[130] Mu N., Wang Y., Li X., Du Z., Wu Y., Su M. (2023). Crotonylated BEX2 interacts with NDP52 and enhances mitophagy to modulate chemotherapeutic agent-induced apoptosis in non-small-cell lung cancer cells. Cell Death Dis..

[131] Abnet C.C., Arnold M., Wei W.Q. (2018). Epidemiology of esophageal squamous cell carcinoma. Gastroenterology.

[132] Lee N.P., Chan C.M., Tung L.N., Wang H.K., Law S. (2018). Tumor xenograft animal models for esophageal squamous cell carcinoma. J. Biomed. Sci..

[133] Sagawa H., Naiki-Ito A., Kato H., Naiki T., Yamashita Y., Suzuki S. (2015). Connexin 32 and luteolin play protective roles in non-alcoholic steatohepatitis development and its related hepatocarcinogenesis in rats. Carcinogenesis.

[134] de Ronde J.J., Lips E.H., Mulder L., Vincent A.D., Wesseling J., Nieuwland M. (2012). SERPINA6, BEX1, AGTR1, SLC26A3, and LAPTM4B are markers of resistance to neoadjuvant chemotherapy in HER2-negative breast cancer. Breast Cancer Res. Treat..

[135] Zhang Z.H., Luan Z.Y., Han F., Chen H.Q., Liu W.B., Liu J.Y. (2019). Diagnostic and prognostic value of the BEX family in lung adenocarcinoma. Oncol. Lett..

